# Supplementary figures and images for: GPCRs show widespread differential mRNA expression and frequent mutation and copy number variation in solid tumors
Source: PLoS Biol. 2019 Nov 25;17(11):e3000434. doi: 10.1371/journal.pbio.3000434 (PMC6901242; doi:10.1371/journal.pbio.3000434)

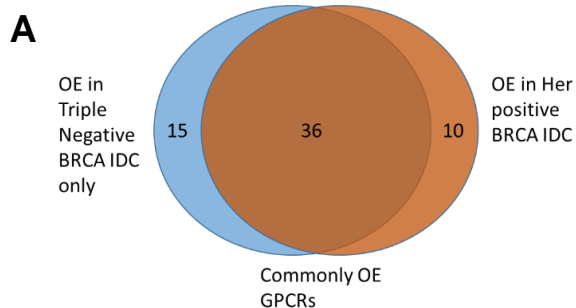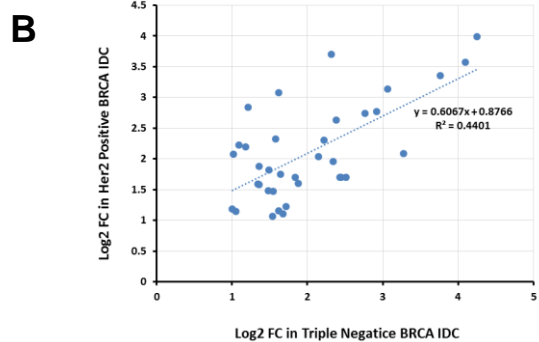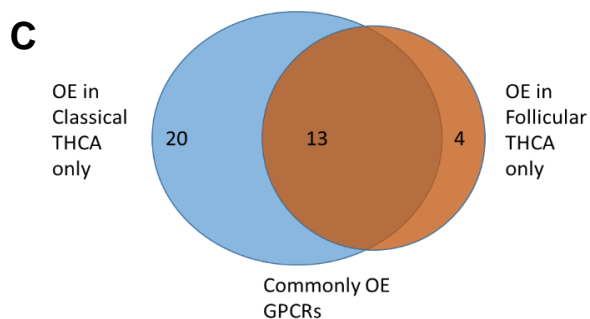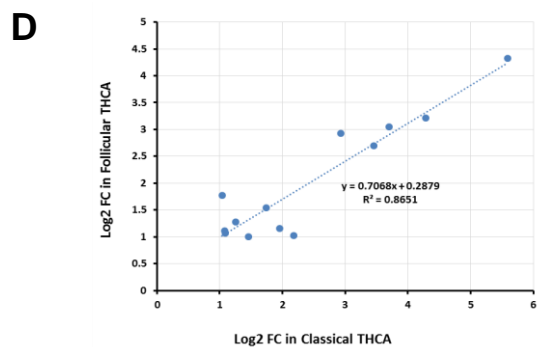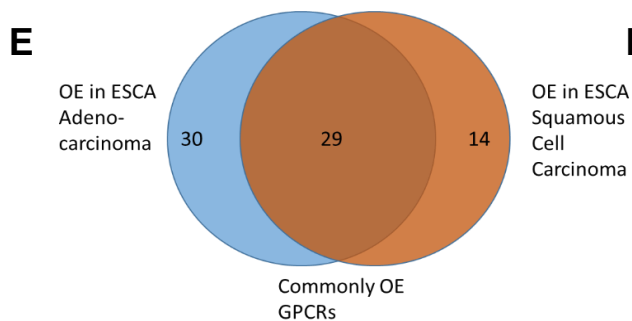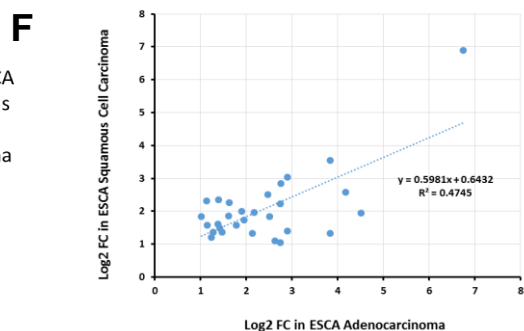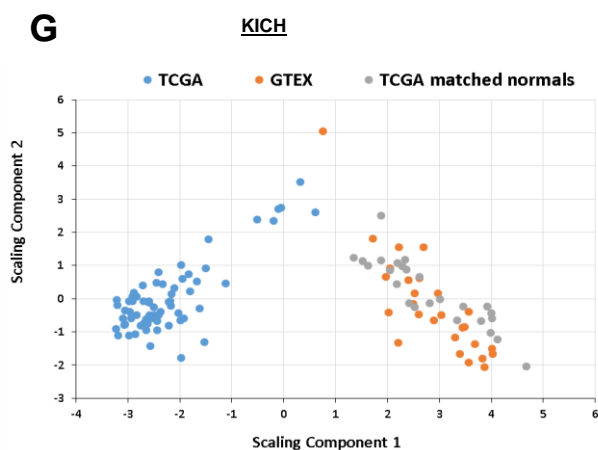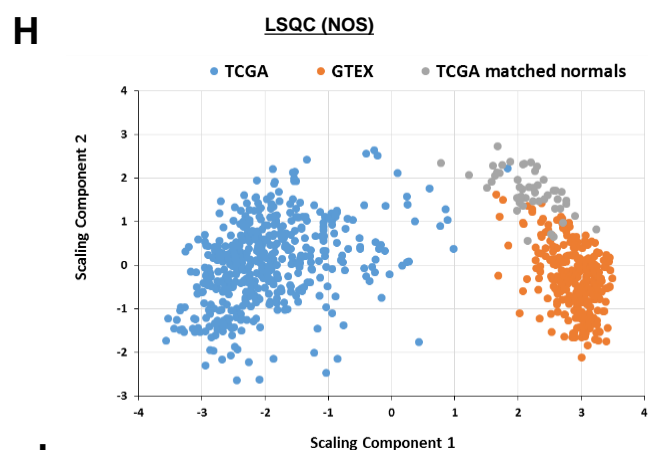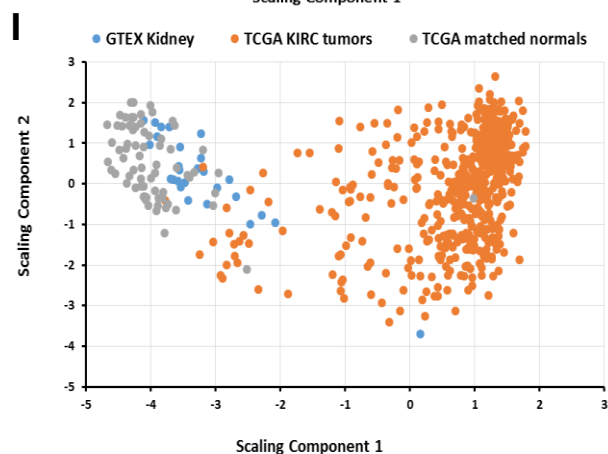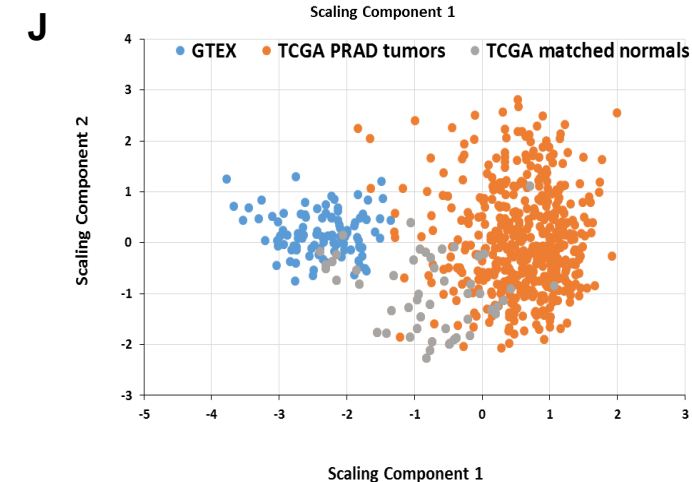

Supplement: S1 Fig — (A–F) DE of GPCRs differs in different cancer subtypes within the same cancer category. (A) The repertoire of overexpressed (OE) GPCRs in Her2-positive and triple-negative BRCA IDC (breast adenocarcinoma, IDC). (B) For commonly OE GPCRs, the correlation of magnitude of fold-changes in expression in each tumor subtype compared to normal breast tissue. (C) The repertoire of OE GPCRs in classical and follicular THCA. (D) For commonly OE GPCRs, the correlation of magnitude of fold-changes in expression in each tumor subtype compared to normal thyroid tissue. (E) The repertoire of OE GPCRs in ESCA adenocarcinoma and squamous cell carcinoma. (F) For commonly OE GPCRs, the correlation of magnitude of fold-changes in expression in each tumor type compared to normal esophageal mucosal tissue. BRCA IDC, either Her2-positive or triple-negative, overexpresses a number of GPCRs. Several of these GPCRs are commonly overexpressed (A), but others are OE in one type but not the other. In general, fold-changes of commonly overexpressed GPCRs correlated among cancer subtypes, but often with some scatter (B). Similar results are found in other tumors (e.g., C–D), showing the degree of overlap of overexpressed GPCRs in classical or follicular THCA. Further, in tumors that occur in the same tissue but with different precursor cells (e.g., squamous cell carcinomas versus adenocarcinomas), the repertoire of differentially expressed GPCRs is distinct. Panels E–F illustrate this for ESCA. Thus, in general, tumor types and subtypes with distinct histological classification possess distinct repertoires and changes in expression of GPCRs. (G–J) Differences between TCGA-matched “normal,” GTEx normal tissue, and tumors (KICH, LSQC [NOS]). MDS plots indicate that in some cases (G, H), TCGA-matched normal and GTEx normal tissue are similar, whereas in others (I, J), LSQC (NOS) and PRAD TCGA matched normal and GTEx normal samples differ, although these differences are smaller than the differences be [file pbio.3000434.s001.pdf]

**A**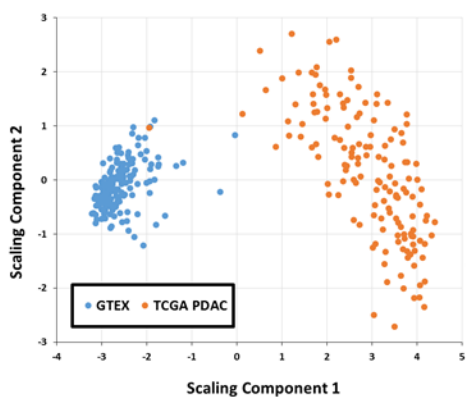**B**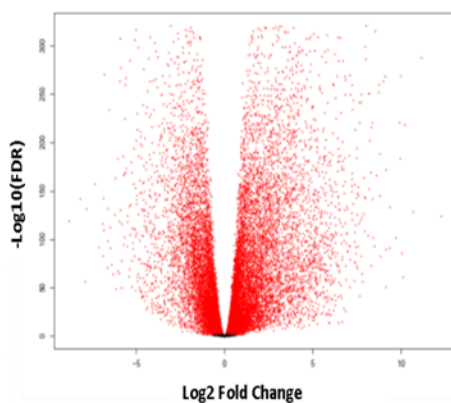**C**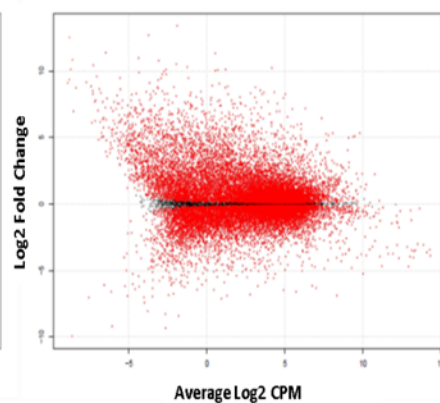**D**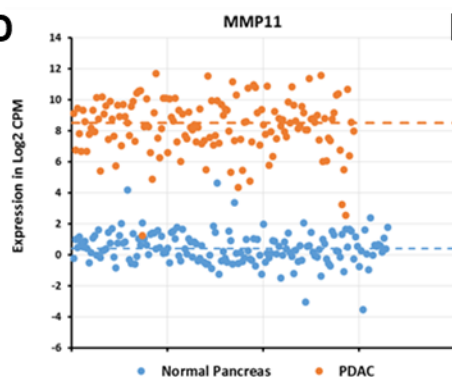**E**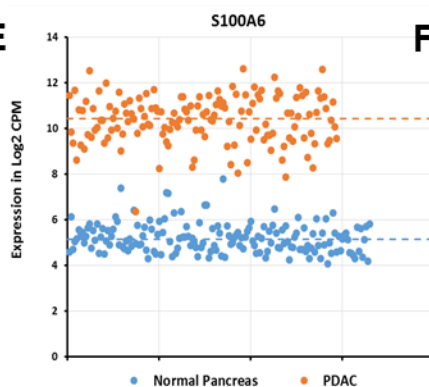**F**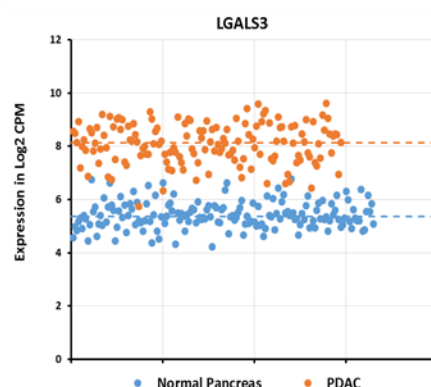**G**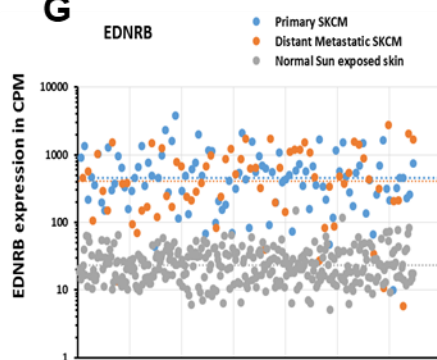**H**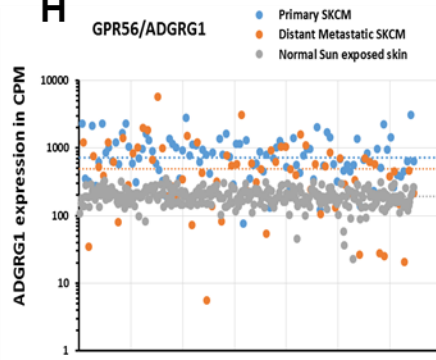**I**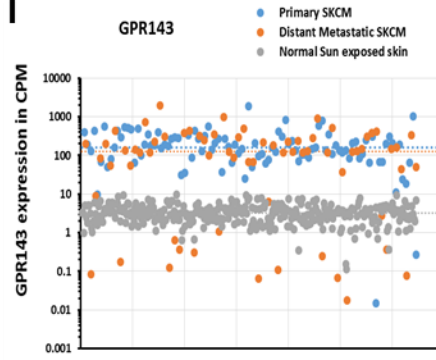**J**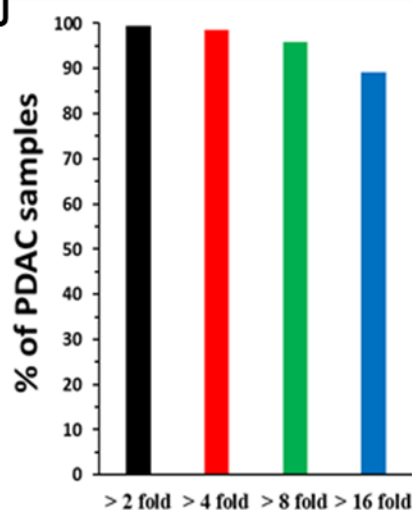

Supplement: S3 Fig — (A) MDS plot of gene expression in normal pancreatic tissue and PDAC tumors. (B) Volcano plot showing significantly differentially expressed genes (FDR < 0.05) in red, with FDR plotted against fold-change. (C) Smear plot showing genes with significant fold-change (red), with fold-change plotted against magnitude of gene expression in CPM. (D–F) Expression of MMP11, S100A6, and LGALS3 in all samples for PDAC and normal pancreas, with medians (dashed lines) also indicated. (G–I) Expression of EDNRB, ADGRG1, and GPR143 in all samples for primary and distant SKCM and normal skin, with medians (dashed lines) also indicated. (J) The fraction of PDAC tumors that express GPRC5A above the indicated thresholds, compared to median expression in normal tissue. MDS plot for part A can be found at https://insellab.github.io/mds_plots. Numerical values for all other plots can be found at https://insellab.github.io/data. (PDF) [file pbio.3000434.s003.pdf]

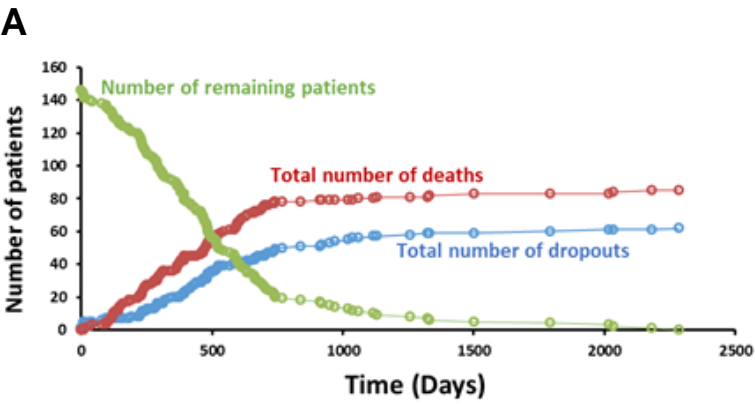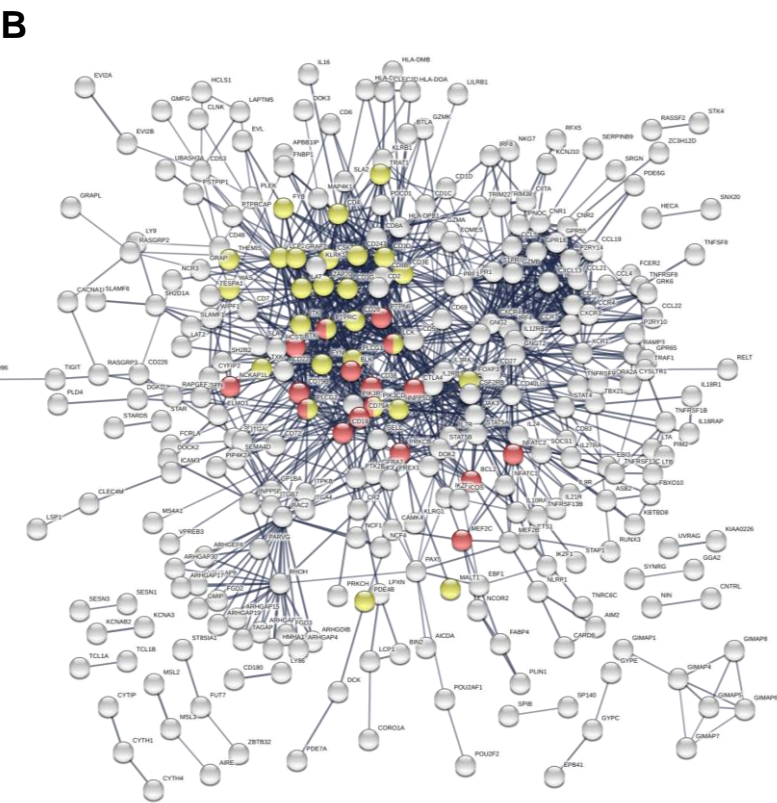

**Yellow: T cell receptor signaling pathway**

**Red: B cell receptor signaling pathway**

Supplement: S4 Fig — (A) The number of patients whose survival was tracked in the TCGA PDAC cohort at each time point, along with the rates of dropout and mortality. (B) Network construction via STRING of the genes the expression of which correlates with that of CCR6, CCR7, CXCR3, and CXCR4. Numerical values for panel A can be found at https://insellab.github.io/data. (PDF) [file pbio.3000434.s004.pdf]

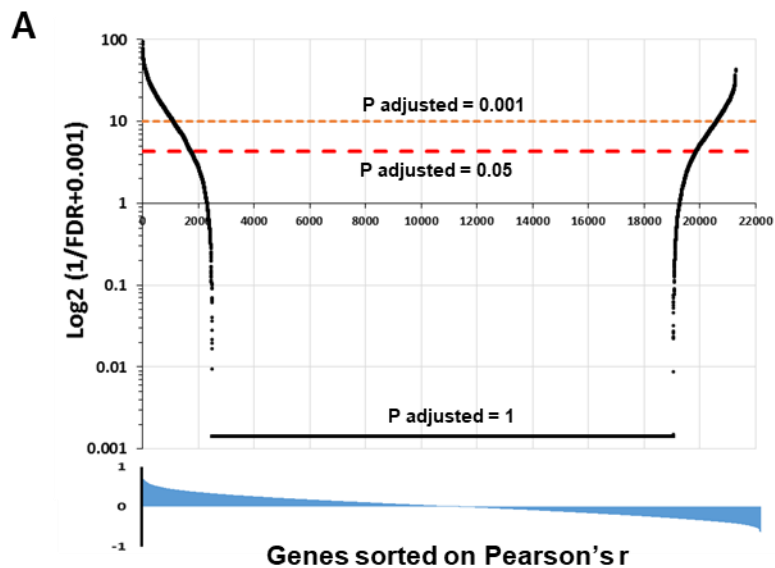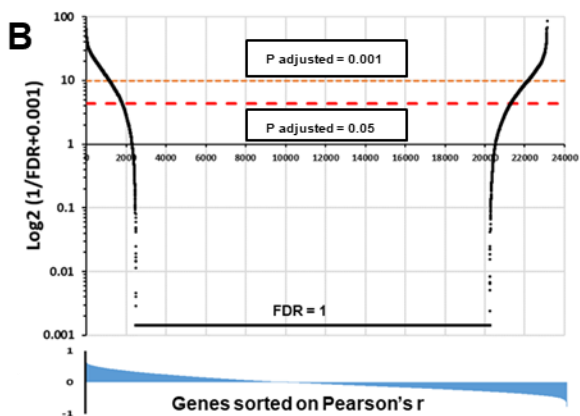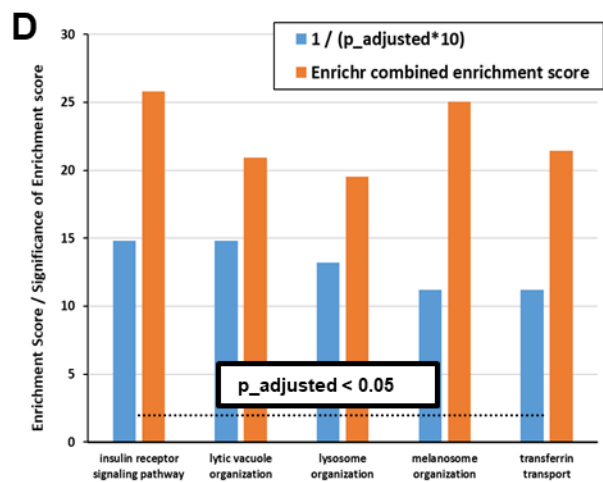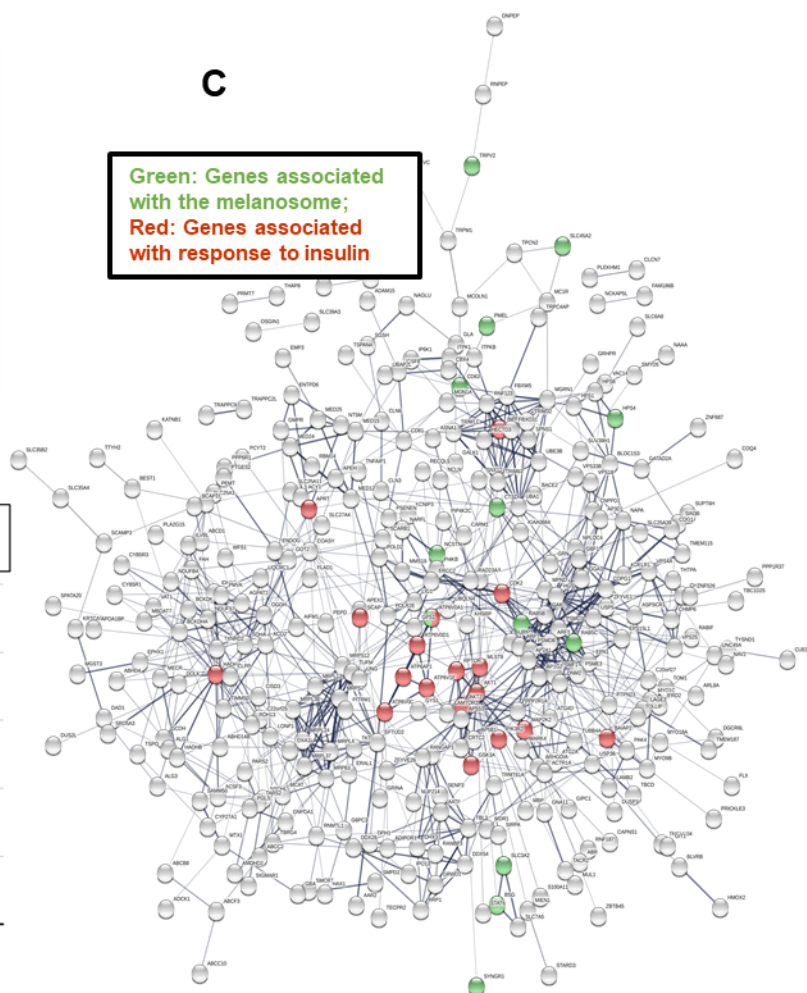

Supplement: S5 Fig — (A) The combined, weighted expression of GPR143, ADGRG1, and EDNRB in SKCM shows positive correlation with expression of a subset of nearly 2,000 genes. (B) Network construction via STRING of the top 500 most strongly correlated genes from (A) illustrating the presence of genes related to the melanosome and to insulin response as examples of cancer-associated pathways in SKCM. (C) Analysis of the 500 most strongly correlated genes via Enrichr shows enrichment of pathways such as transferring signaling, insulin response, etc. among these positively correlated genes. Numerical values for panel C can be found at https://insellab.github.io/data. (PDF) [file pbio.3000434.s005.pdf]

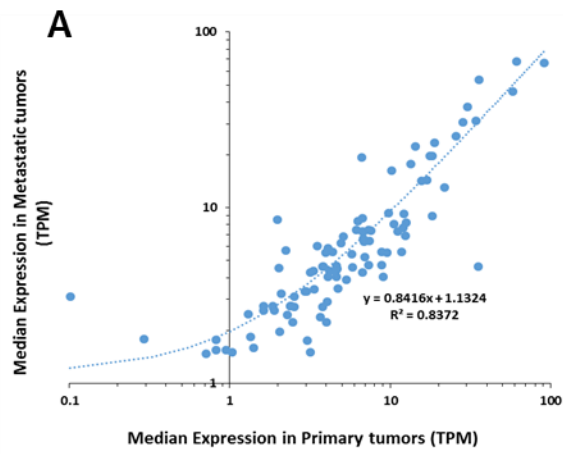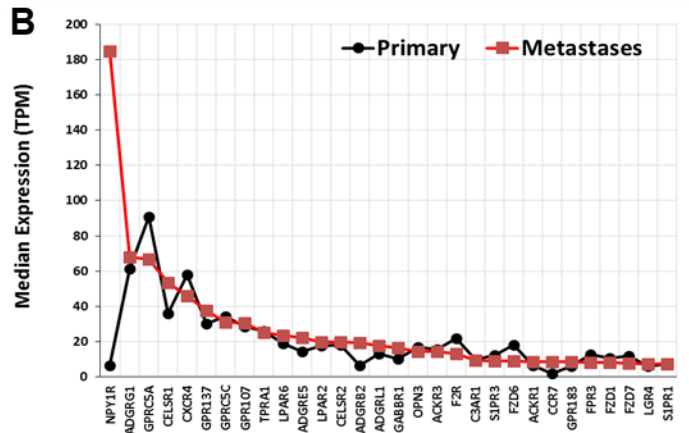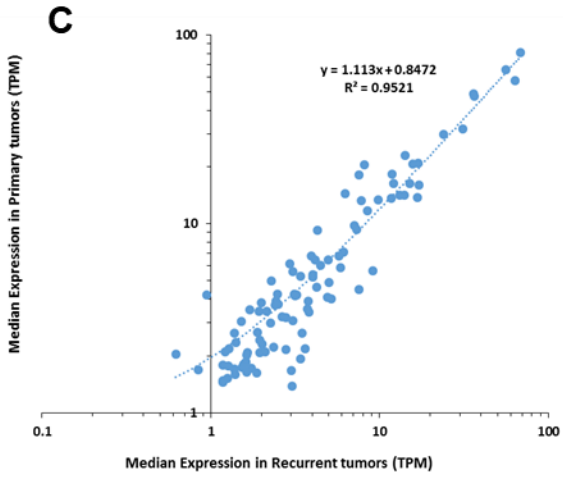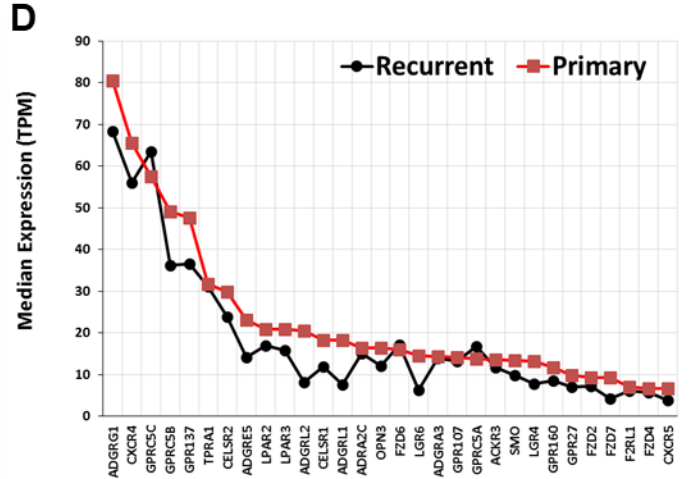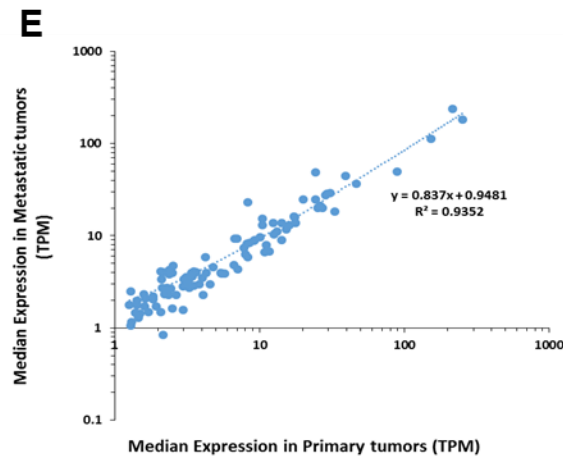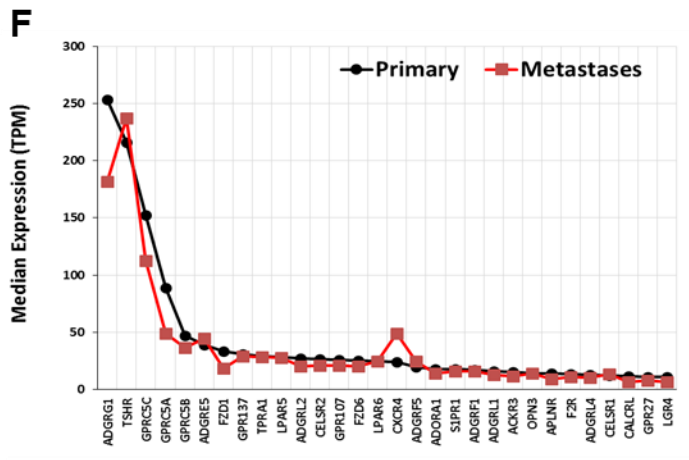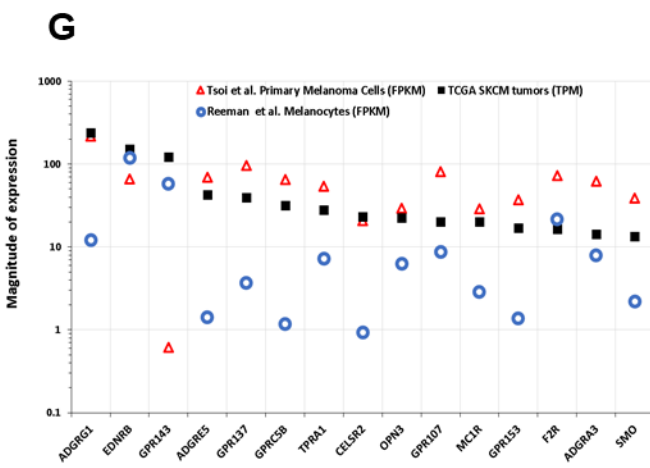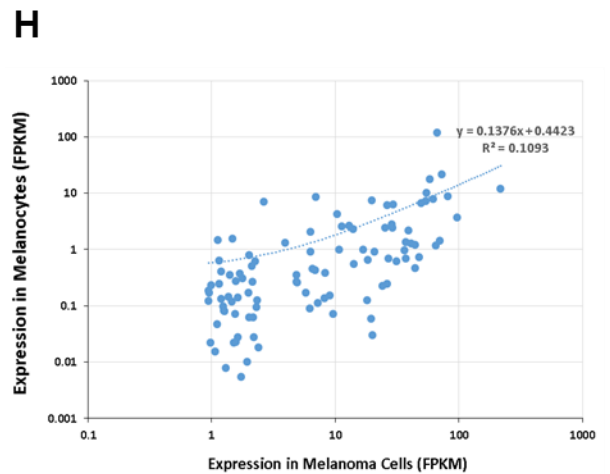

Supplement: S6 Fig — (A–F) GPCR expression in metastatic and recurrent OV, thyroid cancer, and BRCA is similar to that in primary tumors. Most TCGA tumor types have few replicates of metastases or recurrent tumors. However, for those with available data (SKCM, Fig 7E; BRCA, THCA, and OV in this figure, discussed below), we tested whether GPCR expression is similar in primary tumors and metastases and in recurrent tumors. Panels A–F show that recurrent ovarian cancers, metastatic THCA (classical) tumors, and BRCA IDC, respectively, have similar GPCR expression, with identities of expressed GPCRs and magnitude of expression similar to primary tumors. One exception in BRCA IDC was NPY1R, which is more highly expressed in metastases than in primary tumors. All BRCA IDC subtypes were combined (e.g., Her2+, triple negative) for these analyses as there were only 6 metastases, precluding comparison of metastases in the different BRCA IDC subtypes. (A) Correlation of expression of the 100 highest expressed genes in primary OV with that of recurrent tumors. (B) Identity and relative expression of the 30 highest expressed GPCRs in primary and recurrent ovarian tumors. (C) Correlation of expression of the 100 highest expressed genes in primary THCA (classical) compared to that of metastatic tumors. (D) Identity and relative expression of the 30 highest expressed GPCRs in the primary and metastatic thyroid tumors. (E) Correlation of expression of the 100 highest expressed genes in primary and metastatic BRCA (IDC, all types combined), excluding NPY1R, which is much higher expressed in metastatic than primary tumors. (F) Identity and relative expression of the 30 highest GPCRs (including NPY1R) in primary and metastatic BRCA IDC tumors. (G, H) GPCR expression in nondiseased melanocytes differs from that in melanoma cells. (G) GPCR expression in low-passage melanoma cancer cells (data mined from Müller and colleagues [41]) indicates that most highly expressed GPCRs in TCGA tumors are also highly expre [file pbio.3000434.s006.pdf]

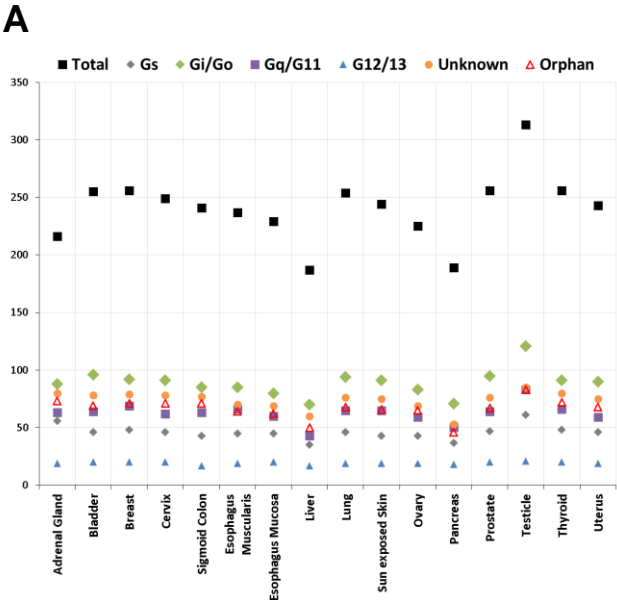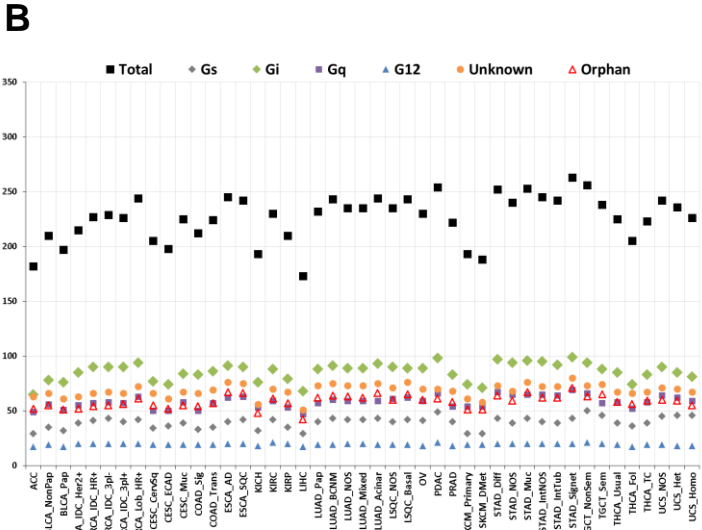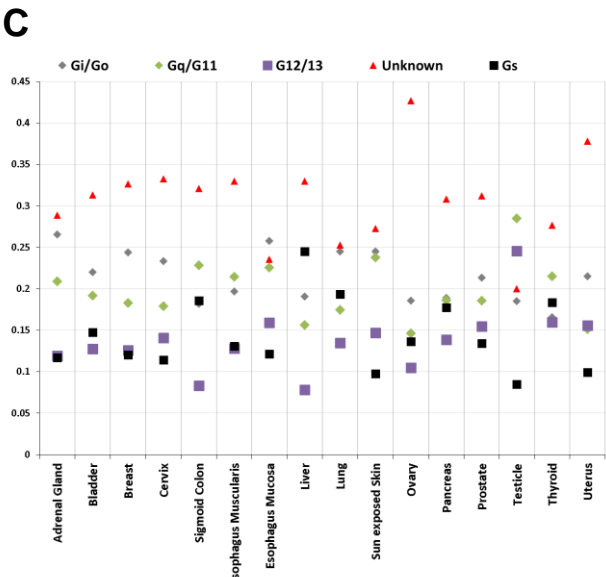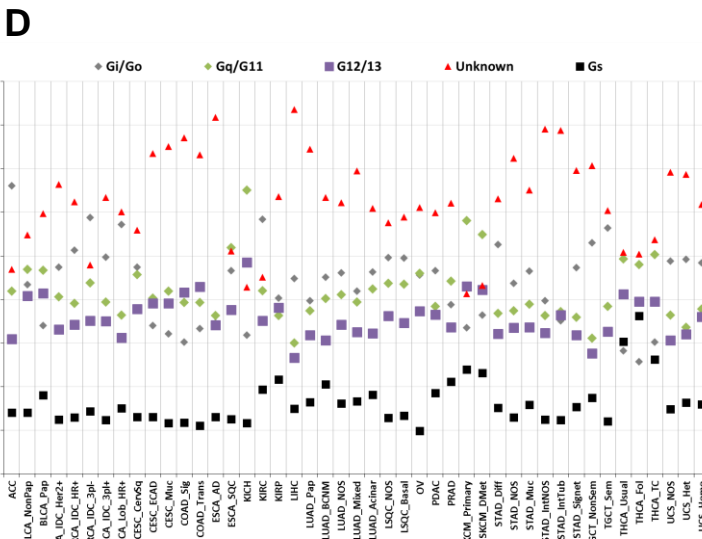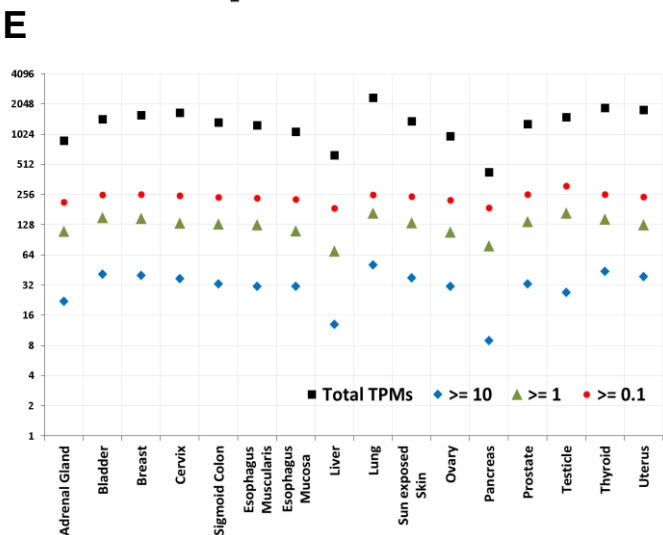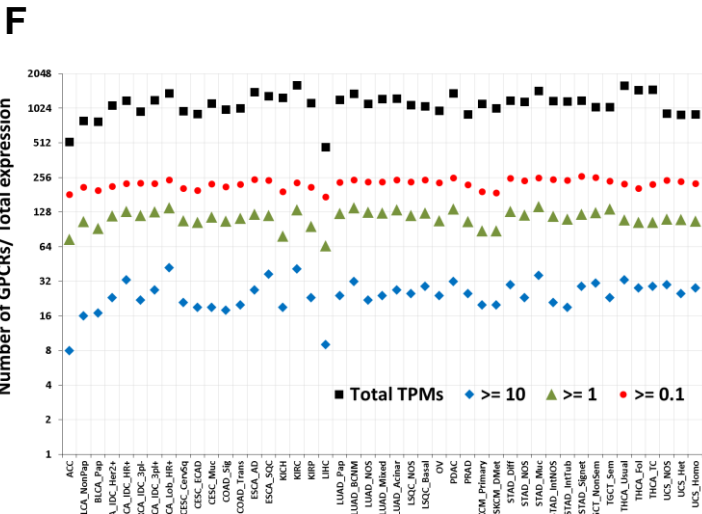

Supplement: S7 Fig — (A, B) The number of GPCRs, orphan GPCRs, and GPCRs that couple to each G protein class in normal tissues (A) and solid tumors (B) and that have ≥0.1 TPM median expression. (C, D) GPCRs that couple to different G proteins compared to the total GPCR expression repertoire in normal tissue (C) and solid tumors (D). (E, F) GPCR expression (TPM) in normal tissue (E) and solid tumors (F), and the number of GPCRs detected at different thresholds of expression. GPCRs typically account for <0.1% of the tissue and tumor transcriptomes. Numerical values for all panels can be found at https://insellab.github.io/data. (PDF) [file pbio.3000434.s007.pdf]

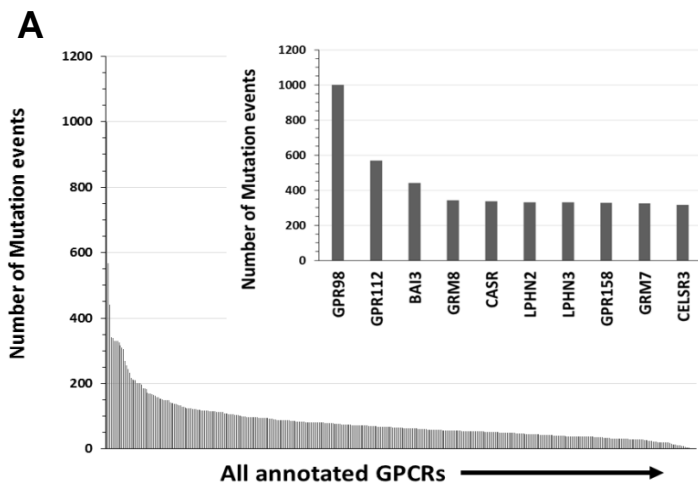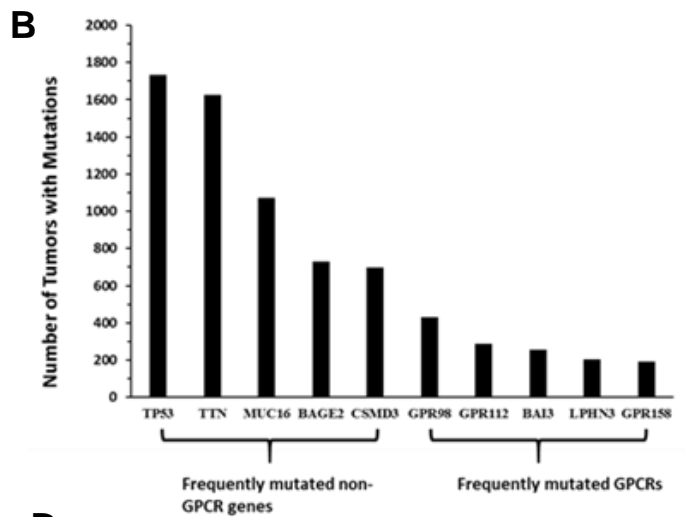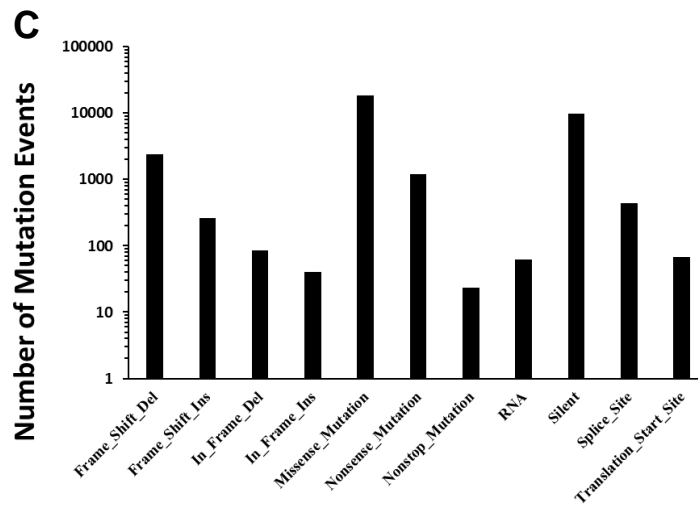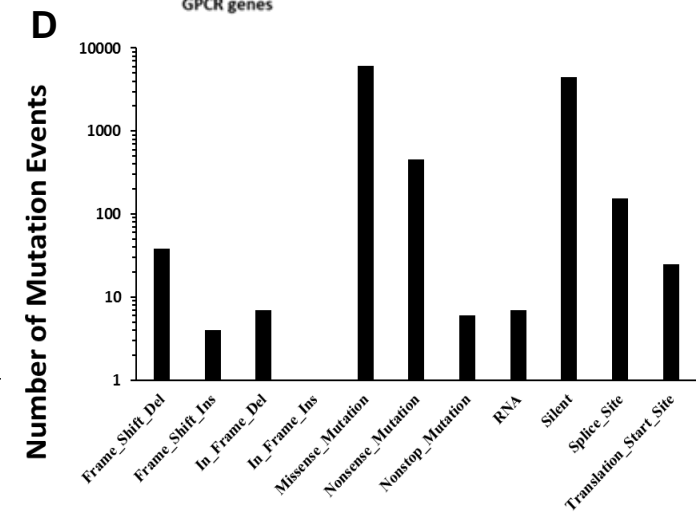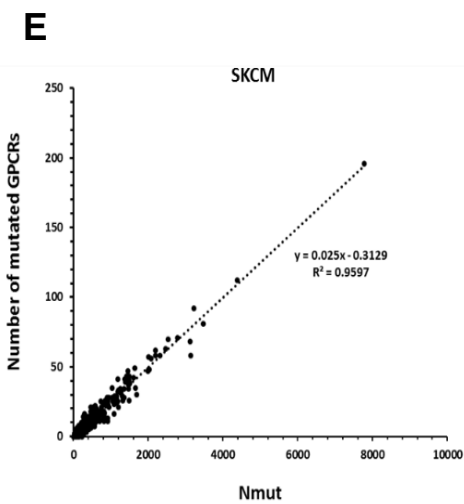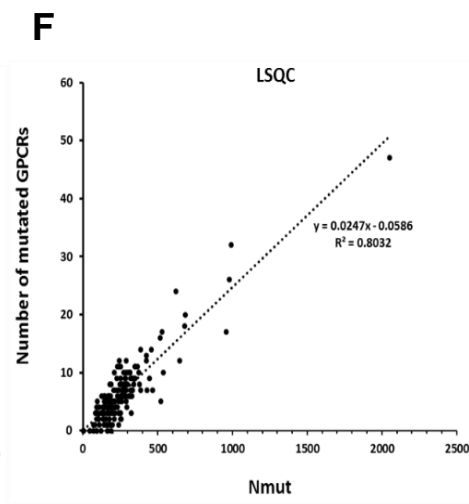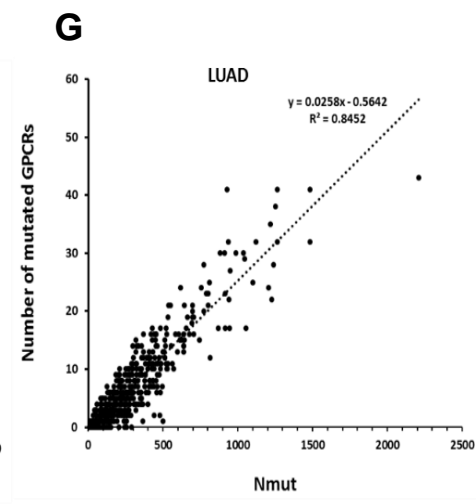

Supplement: S8 Fig — (A, B) Missense mutations are the most frequent type of nonsilent mutation of GPCRs in TCGA tumors, with GPR98/ADGRV1 the most frequently mutated GPCR. (A) The number of each type of somatic mutation for GPCRs in TCGA tumors surveyed (n = 5,103 tumors with 32,727 somatic mutational events in GPCRs. (B) The same analysis for SKCM, which has the highest number (11,348) of GPCR mutational events, i.e., more than one-third of all somatic mutation events but only approximately 9% of TCGA samples. S5 Table lists the total number of mutation events for the most commonly mutated GPCRs; a complete list is provided in S1 Table. GPCRs with the most frequent mutation events are also mutated in the largest number of tumors (Fig 10A). In the 5,103 TCGA tumors surveyed, missense mutations are the most frequent type of nonsilent GPCR mutation, occurring approximately 10-fold more frequently than frameshift deletions, the next most common type of nonsilent mutation. Missense mutations were the most common type of mutational event for GPCRs in all cancer types except LIHC, which had a high frequency of frameshift deletions (n = 1,875 events), with GPR98 the most frequently so mutated (n = 79 events). (C) The number of somatic mutation events in all TCGA tumors for all annotated GPCRs. Inset: the number of mutation events for the 10 most frequently mutated GPCRs. These data mirror those in Fig 10 that show the number of tumors in TCGA that possess somatic, nonsilent mutations to GPCRs. (D) The most frequently mutated GPCR and non-GPCR genes in solid tumors: the number of tumors across all tumor types surveyed with somatic nonsilent mutations for the genes indicated. (E–G) The number of mutated GPCRs in a tumor scales linearly with Nmut, the number of mutated genes per tumor genome. For SKCM, LUAD, and LSQC, their number of mutated GPCRs increases linearly with Nmut. This linear relationship is found with other tumor types and is nearly identical among tumor types, implying a general p [file pbio.3000434.s008.pdf]

**A**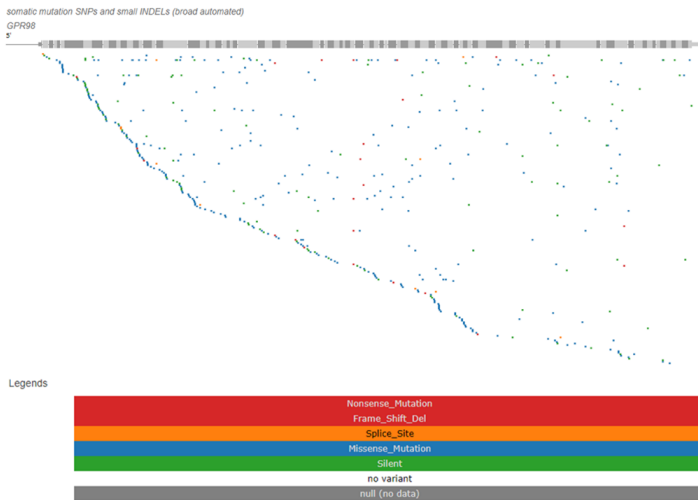**B**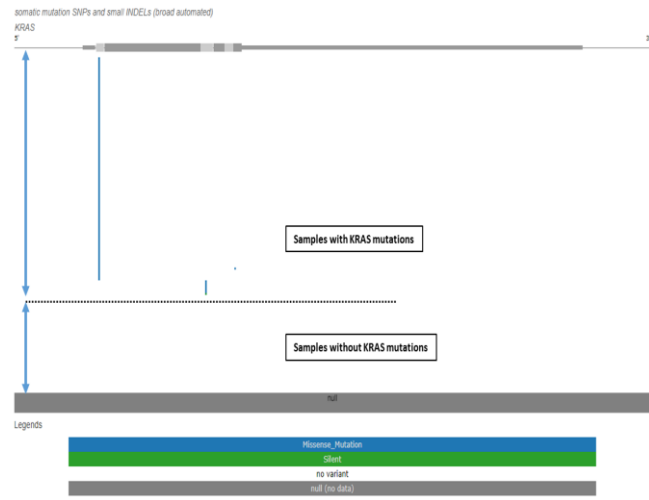**C**

Primary SKCM

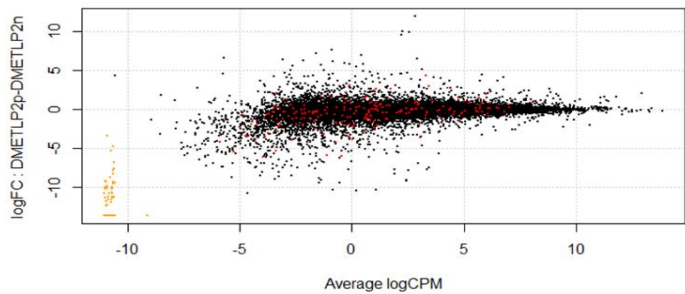**D**

Distant Metastatic SKCM

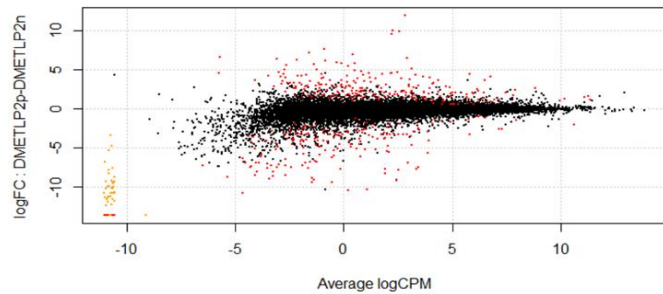**E**

Primary SKCM

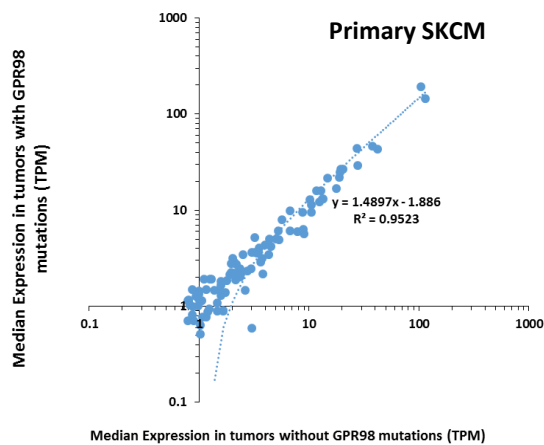**F**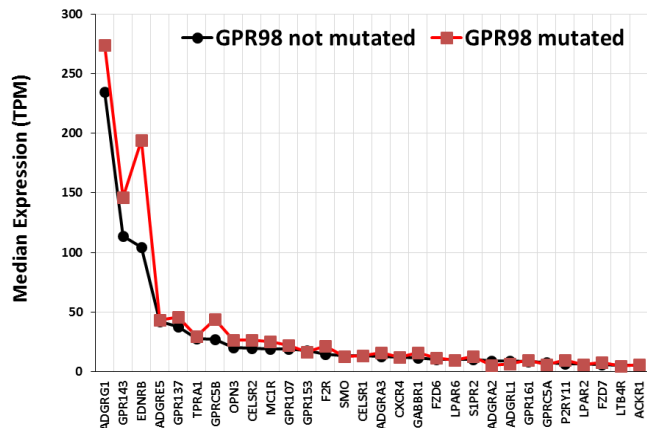**G**

Distant SKCM metastases

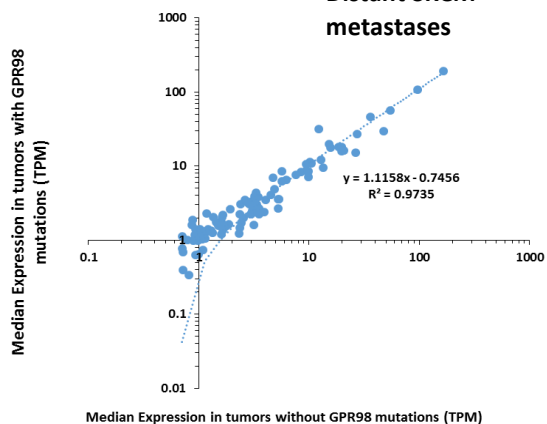**H**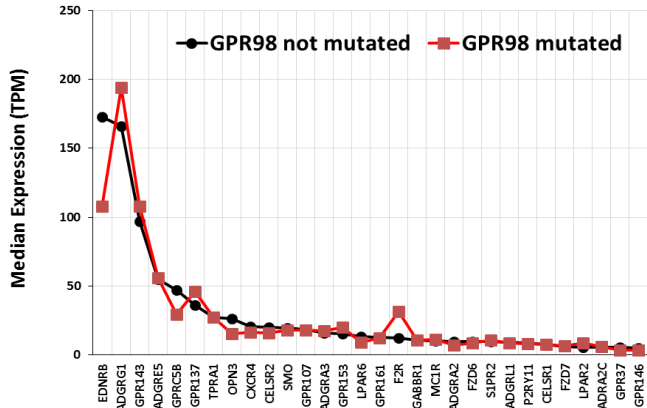

Supplement: S9 Fig — (A, B) Location of silent and nonsilent mutations of GPR98/ADGRV1 in TCGA SKCM tumors (A) and KRAS mutations in PAAD tumors (B) accessed via Xena (xena.ucsc.edu). Data are shown for 204/472 SKCM samples in which GPR98 has silent or nonsilent mutations. Introns are not included; hence, the figure shows exonic locations of mutations; 132/186 PAAD samples had somatic KRAS mutations. Vertical gray bars indicate exons. Mutations of GPR98/ADGRV1 are distributed along the length of the gene and are not enriched at specific locations or exons. Thus, large portions of GPR98 represent mutational hotspots, findings that contrast with what occurs in driver mutations such as KRAS, in which mutations at specific sites and specific exons result in gain of function or loss of function, respectively. S9B Fig shows that virtually all somatic (almost exclusively missense) mutations occur at exon 2 (resulting in an oncogenic KRAS) in PAAD, in contrast with the range of mutations in SKCM for GPR98, GPR112, and other GPCR genes. The distribution of mutations along gene length is a feature of GPCR mutations in other cancers as well (e.g., BLCA, LUAD). (C, D) For primary (C) and distant metastatic (D) SKCM samples, relatively few genes show DE if one compares samples with or without LPHN2/ADGRL2 mutations. Fewer than 100 genes increase or decrease >2-fold (with FDR < 0.05 and >1 TPM median expression). Lists of DE genes for each case are provided in S2 Table. Red dots correspond to DE genes with FDR < 0.05. Similar data occur for other frequently mutated GPCRs (e.g., GPR98) in SKCM and in LUAD and BLCA. (E, G) Correlation of GPCR expression (n = 100 highest expressed GPCRs) in primary and distant metastatic SKCM tumors, respectively, with GPR98 somatic nonsilent mutations compared to tumors without GPR98 mutation. (F, H) The identities and median expression (TPM) of the 30 highest-expressed GPCRs in primary and distant metastatic SKCM tumors, respectively, for tumors with or without GPR98 [file pbio.3000434.s009.pdf]

**A**

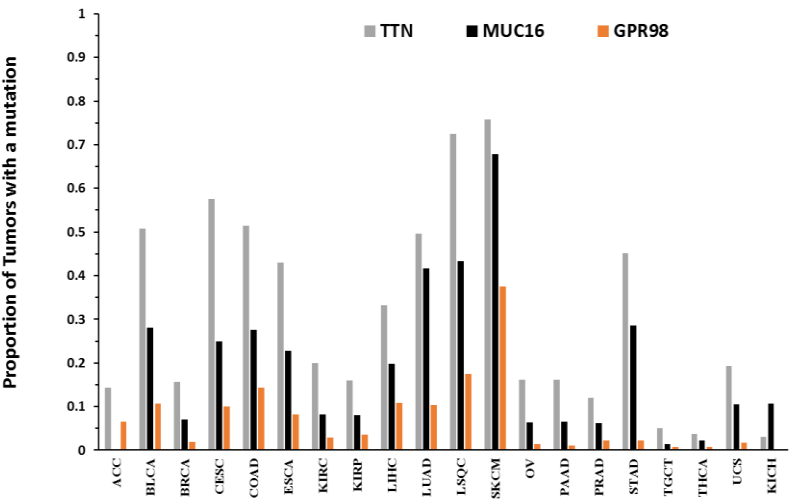

**B**

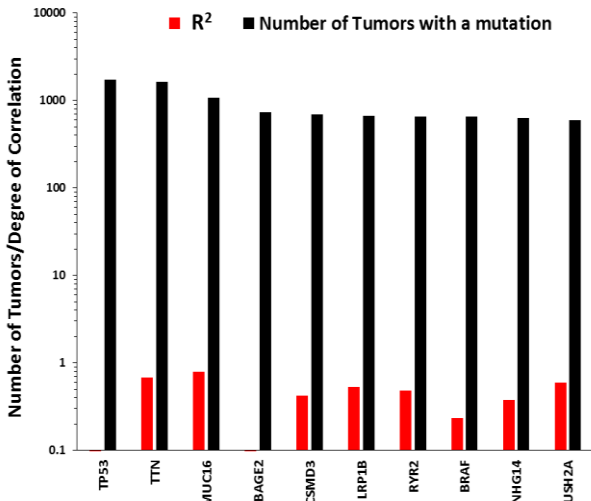

**C**

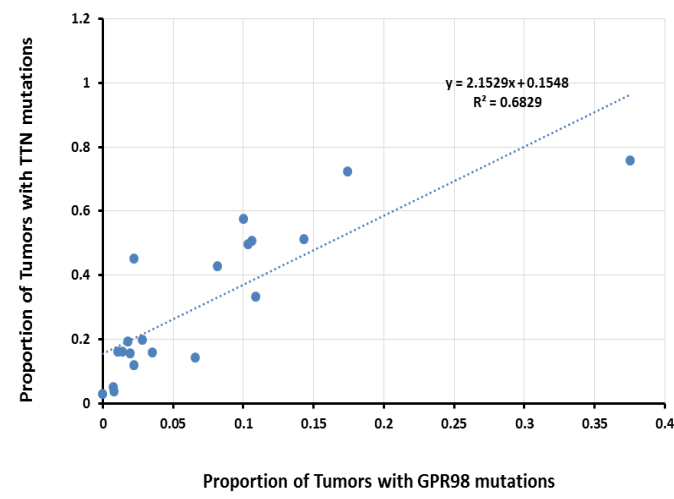

**D**

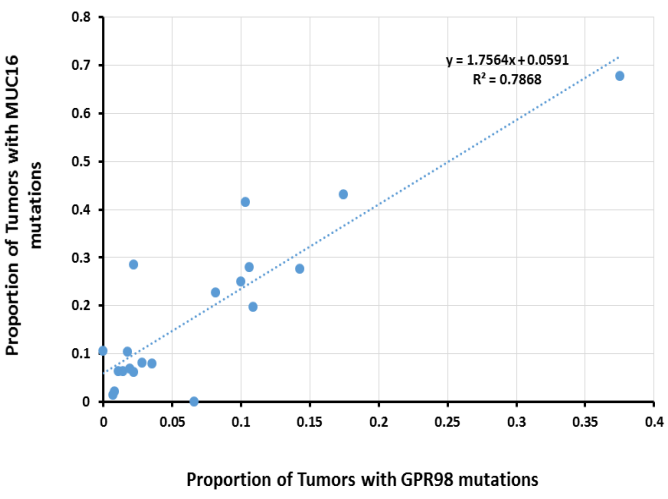

**E**

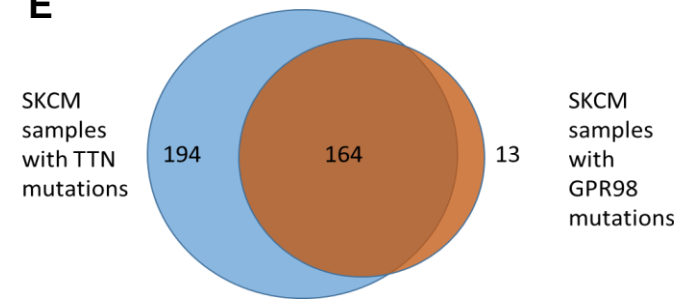

**F**

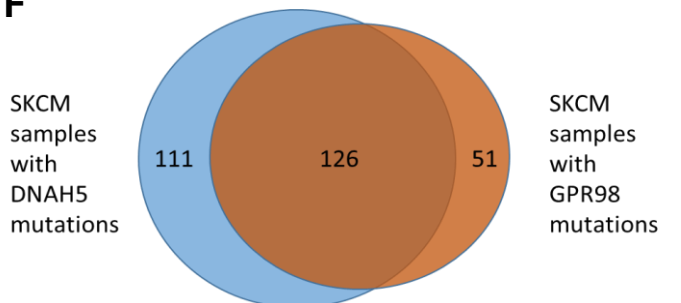

**G**

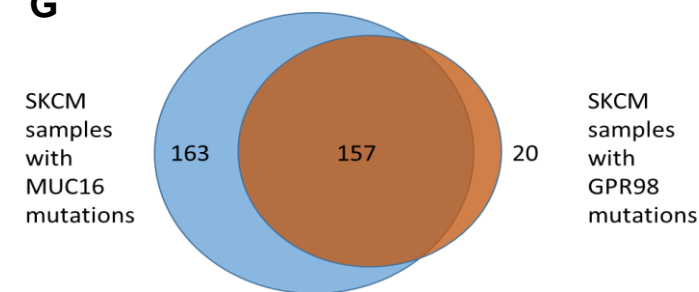

Supplement: S10 Fig — (A–D) Mutations in GPR98 are frequently accompanied by mutations in other genes. The proportion of tumors possessing somatic, nonsilent mutations in GPR98 correlates with that of several other frequently mutated genes. Tumor types that show a high frequency of mutations to genes such as TTN and MUC16 also typically show a high frequency of GPR98 mutations and vice versa for tumors with infrequent mutations to these genes. TTN and MUC16 form a group of genes that, along with GPR98, are frequently mutated across a range of tumors. (A) The proportion of tumors in each TCGA tumor type that show mutations in TTN, MUC16, and GPR98. (B) The correlation between the proportions of tumor samples possessing TTN mutations and GPR98 mutations for the 20 TCGA tumor types shown above. (C) The same data for mutations in MUC16 versus GPR98 mutations. (E, F, G) Tumors with GPR98 mutations frequently have mutations in other frequently mutated genes. SKCM tumors (n = 472) have a high frequency of TTN, MUC16, and DNAH5 somatic, nonsilent mutations. Most SKCM tumors with somatic, nonsilent mutations to GPR98 also have mutations in these other genes. Numerical values for panels A–D can be found at https://insellab.github.io/data. (PDF) [file pbio.3000434.s010.pdf]

A

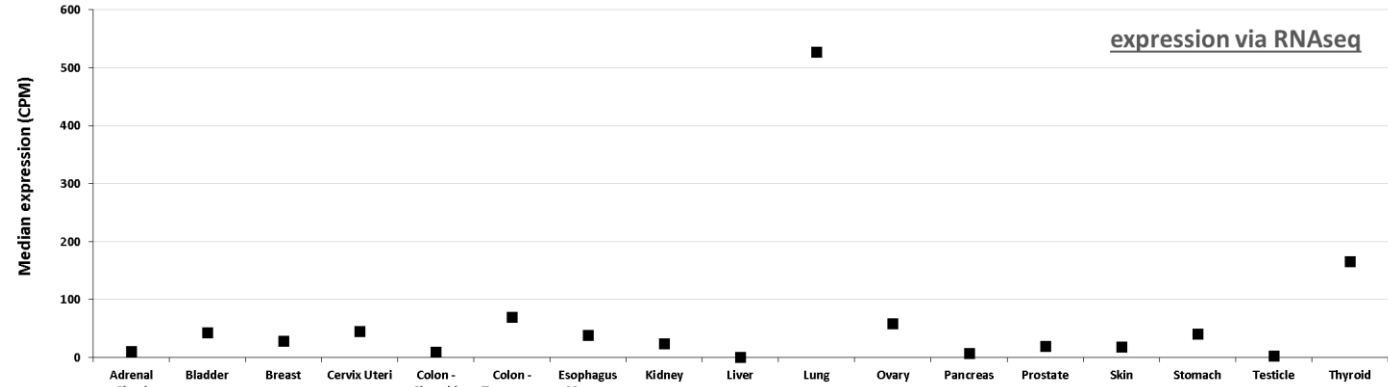

B

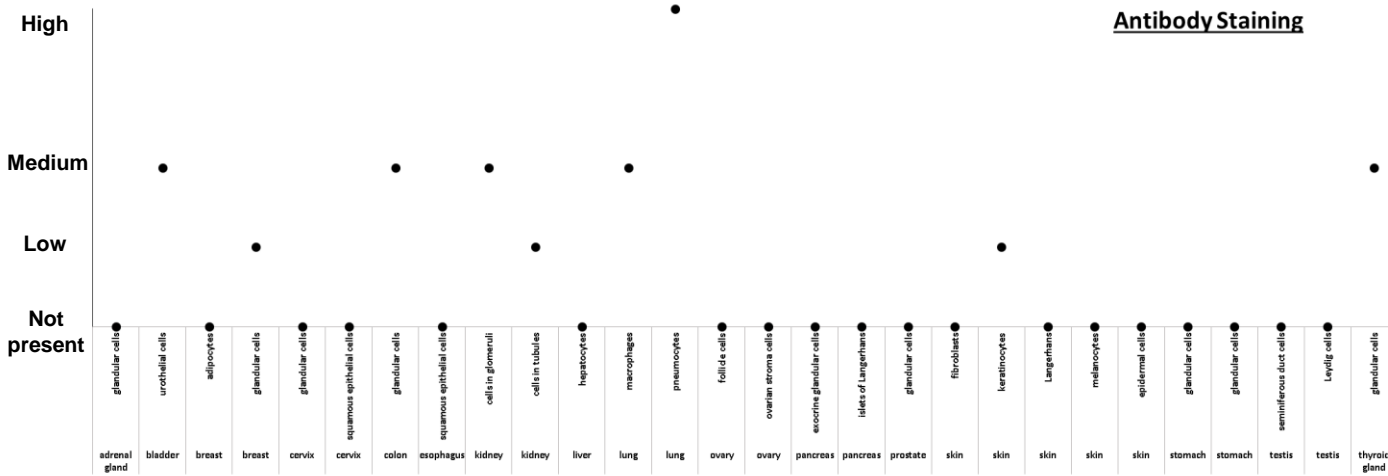

C

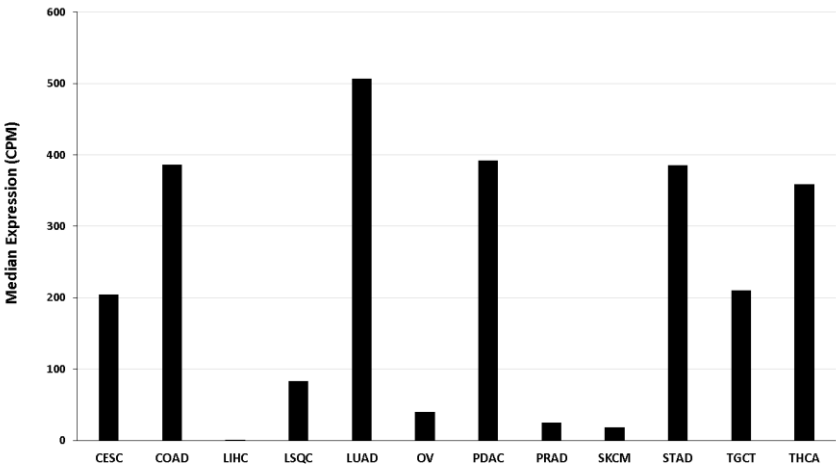

D

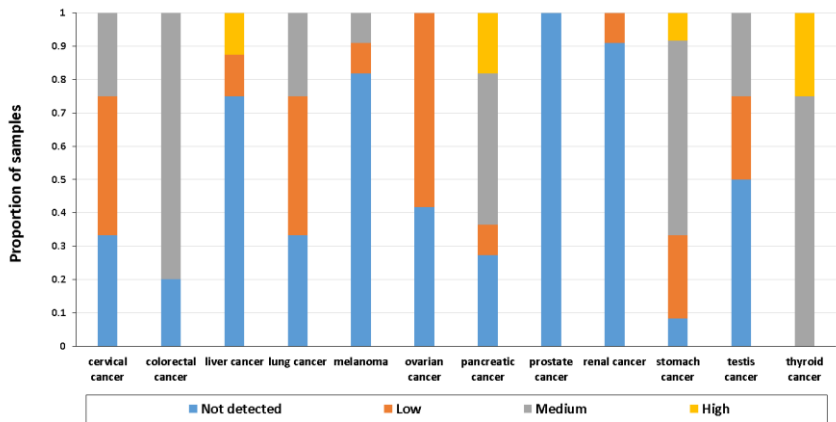

Supplement: S11 Fig — (A) GPRC5A mRNA expression in normal tissue. Median normalized mRNA expression from GTEx, in CPM of GPRC5A in normal tissues profiled by RNA-seq. (B) GPRC5A protein expression in normal tissue. Protein abundance (by immunohistochemistry from the human protein atlas; https://www.proteinatlas.org/) of GPRC5A in normal tissue. (C) GPRC5A mRNA expression in tumors. Median normalized mRNA expression (in CPM in TCGA) of GPRC5A in tumors profiled by RNA-seq. For TCGA tumor types with multiple subtypes (e.g., LUAD), values are the median for all subtypes of the tumor type to facilitate comparison with protein atlas data, in which TCGA tumor types are not separated into subtypes. (D) GPRC5A protein expression in tumor tissue. Protein abundance (by immunohistochemistry from the human protein atlas; proteinatlas.org) of GPRC5A in a range of tumor types. Data are provided for multiple replicates at https://www.proteinatlas.org/ENSG00000013588-GPRC5A/pathology; the proportion of samples staining at different intensity levels are shown. Values plotted for all panels are available at https://insellab.github.io/data. Data from the human protein atlas were downloaded from https://www.proteinatlas.org/about/download (bulleted items 1 and 2), and data for GPRC5A were extracted from the relevant files. (PDF) [file pbio.3000434.s011.pdf]

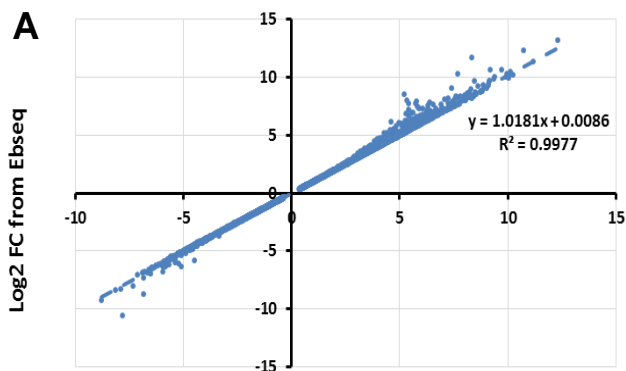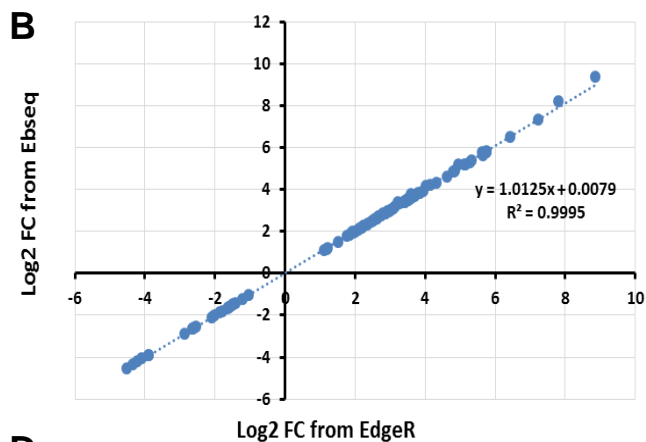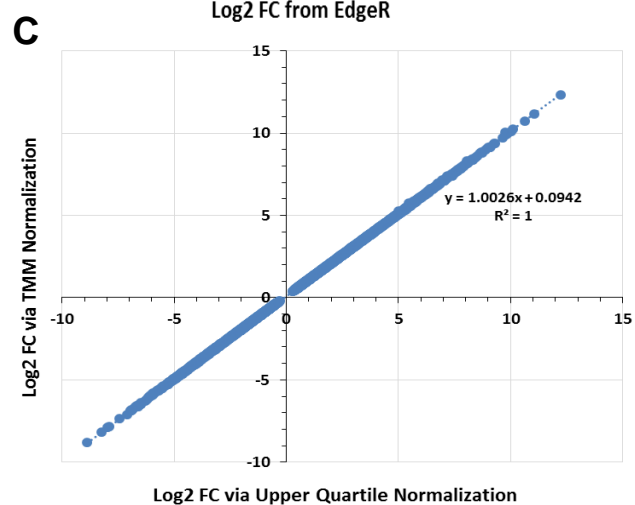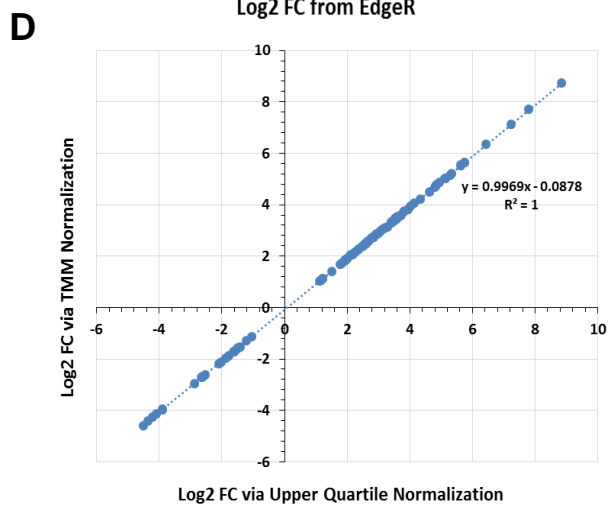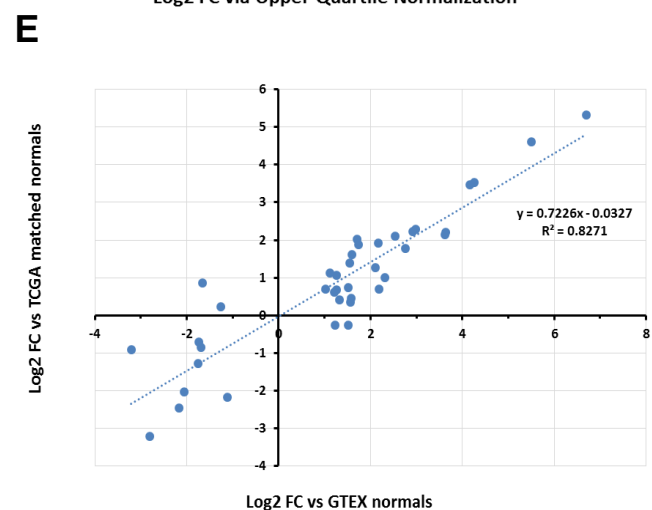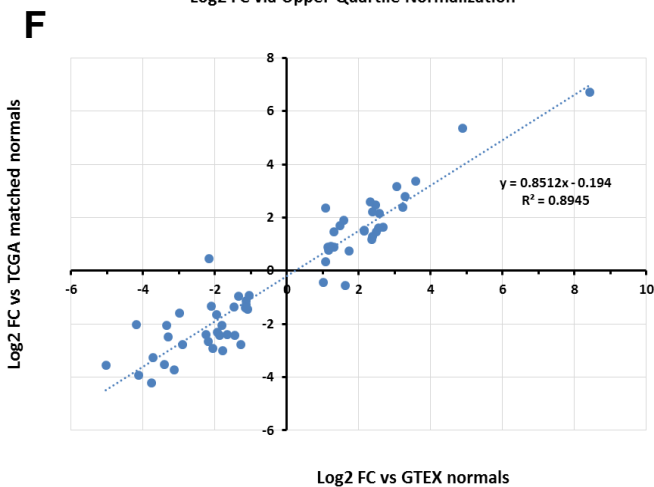

Supplement: S12 Fig — (A, B) EBseq and EdgeR yield similar results for DE analysis. (A) Comparison of fold-changes determined via EdgeR for the 10,000 genes with lowest FDRs (i.e., the most significantly altered genes), compared to the fold-changes for the same genes determined via Ebseq, using data for PDAC tumors compared to normal pancreatic tissue. (B) The same comparison (between EBseq and EdgeR) for the 100 GPCRs with lowest FDRs calculated via EdgeR. Dashed lines indicate linear fits. The two methods make different statistical assumptions but yield similar results, especially for GPCRs. We chose to use EdgeR rather than Ebseq based on the much lower processing times in EdgeR for the large files we generated. These results, along with the similarity of DE analysis between upper-quartile and TMM normalized data, helps allay concerns regarding the validity of this analysis stemming from the large numbers of DE genes found when comparing tumor and normal samples. (C, D) TMM and upper-quartile normalization in EdgeR yield similar results for DE analysis. (C) Comparison of fold-changes determined via EdgeR for the 10,000 genes with lowest FDRs (i.e., the most significantly altered genes) compared to fold-changes for the same genes determined via Ebseq, using data for PDAC tumors compared to normal pancreatic tissue. (D) The same comparison for the 100 GPCRs with lowest FDRs calculated via EdgeR. Dashed lines indicate linear fits. (E, F) DE analysis of fold-changes for GPCRs identified as meaningfully increased or decreased expression in KICH (E) and LSQC (NOS); (F) is similar (especially for highly expressed GPCRs with high fold-change) whether one compares these tumors with GTEx normal tissue or TCGA-matched normal samples. Numerical values for all figure panels can be found at https://insellab.github.io/data. (PDF) [file pbio.3000434.s012.pdf]

SUPPLEMENTARY FIGURE 13

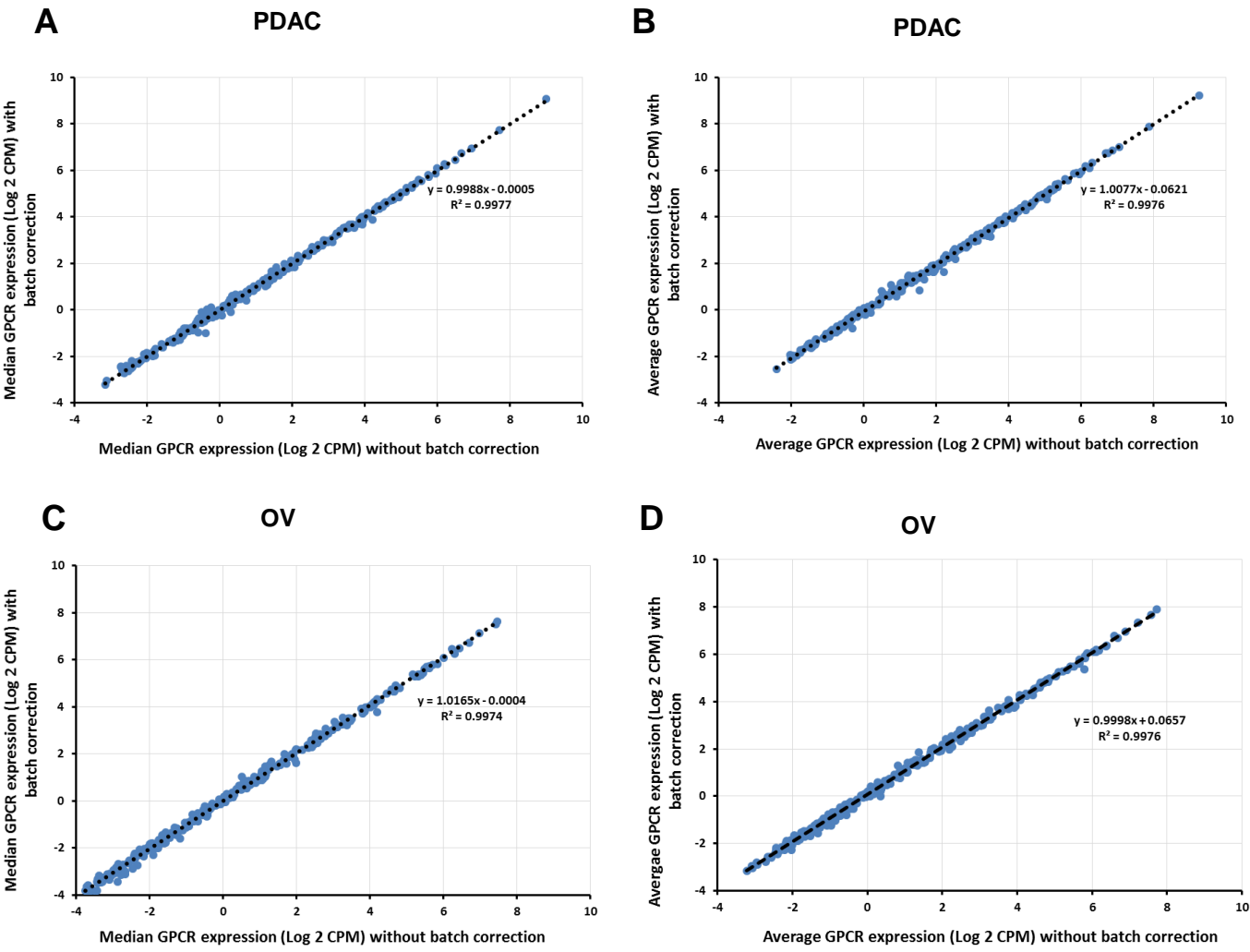

Supplement: S13 Fig — (A, B) Correlation of median (a) and average (b) expression of GPCRs in TCGA PDAC samples, with and without batch corrections performed for TCGA plate ID. (C, D) Correlation of median (c) and average (d) expression of GPCRs in OV (TCGA “Ovarian Cancer”) samples, with and without batch corrections performed for TCGA plate ID. Numerical values for all figure panels, showing corresponding GPCR expression before and after batch corrections, can be found at https://insellab.github.io/data. (PDF) [file pbio.3000434.s013.pdf]
