# Supplementary material for: GPCRs show widespread differential mRNA expression and frequent mutation and copy number variation in solid tumors
Source: PLoS Biol. 2019 Nov 25;17(11):e3000434. doi: 10.1371/journal.pbio.3000434 (PMC6901242; doi:10.1371/journal.pbio.3000434)
Supplement: S2 Fig — (PDF) [file pbio.3000434.s002.pdf]

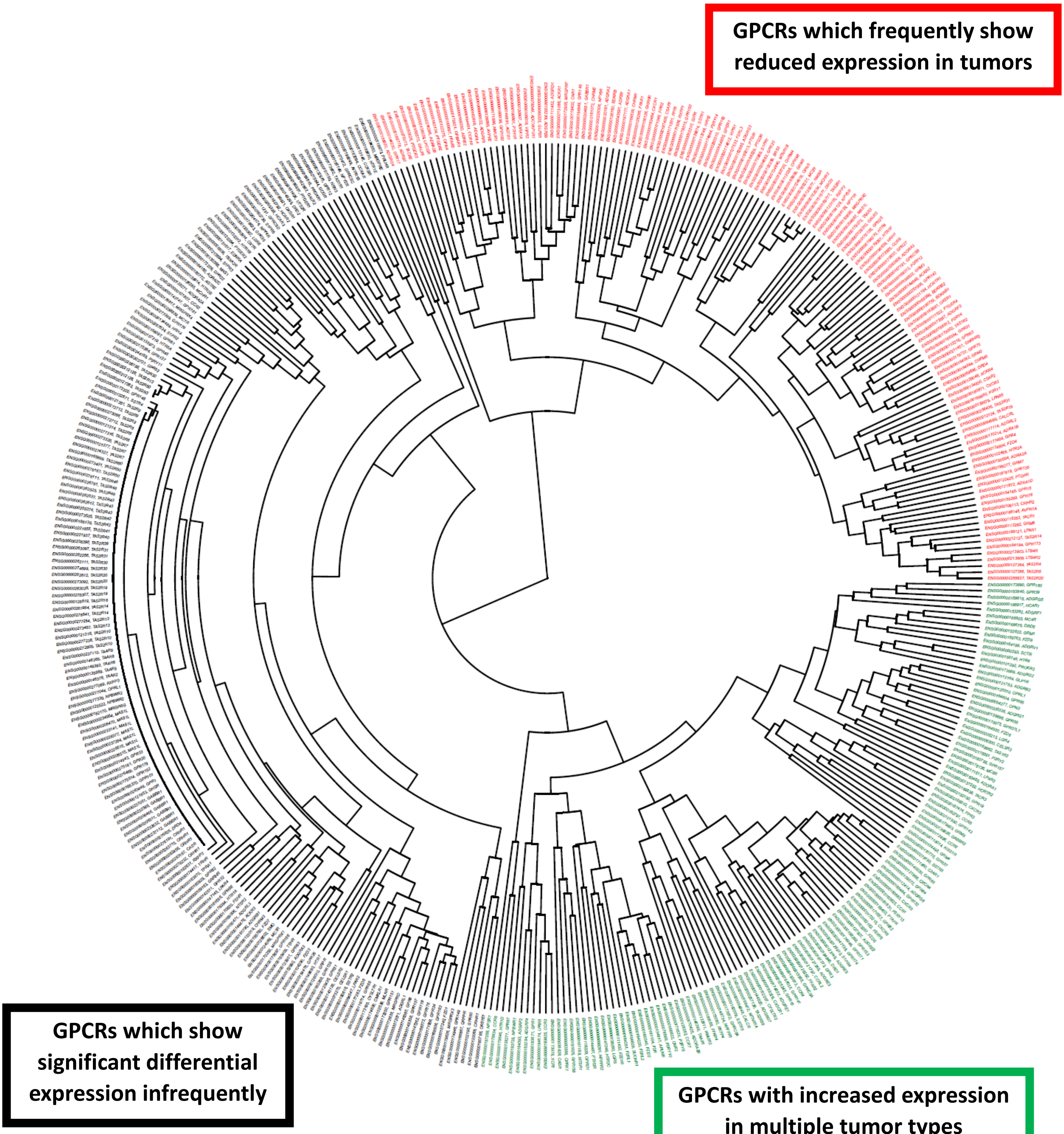
GPCRs which frequently show reduced expression in tumors
GPCRs which show significant differential expression infrequently
GPCRs with increased expression in multiple tumor types
