# Supplementary material for: GPCRs show widespread differential mRNA expression and frequent mutation and copy number variation in solid tumors
Source: PLoS Biol. 2019 Nov 25;17(11):e3000434. doi: 10.1371/journal.pbio.3000434 (PMC6901242; doi:10.1371/journal.pbio.3000434)
Supplement: S3 Table — Mutation and CNV data were obtained from xena.ucsc.edu. For mutations, data were generated at the Broad Institute Sequencing Center, except for *: Baylor College of Medicine Sequencing Center and #: Washington University Sequencing Center. Mutation data were generated via automated pipelines from the respective sources, hosted at xena.ucsc.edu. (DOCX) [file pbio.3000434.s016.docx]

**S3Table**. **The types of cancers and number of replicates from TCGA surveyed for GPCR mutations and CNV**. Mutation and CNV data were obtained from *xena.ucsc.edu*. For mutations, data were generated at the Broad institute sequencing center, except for * Baylor College of Medicine Sequencing Center and # Washington University Sequencing Center. Mutation data were generated via automated pipelines from the respective sources, hosted at *xena.ucsc.edu*.

*
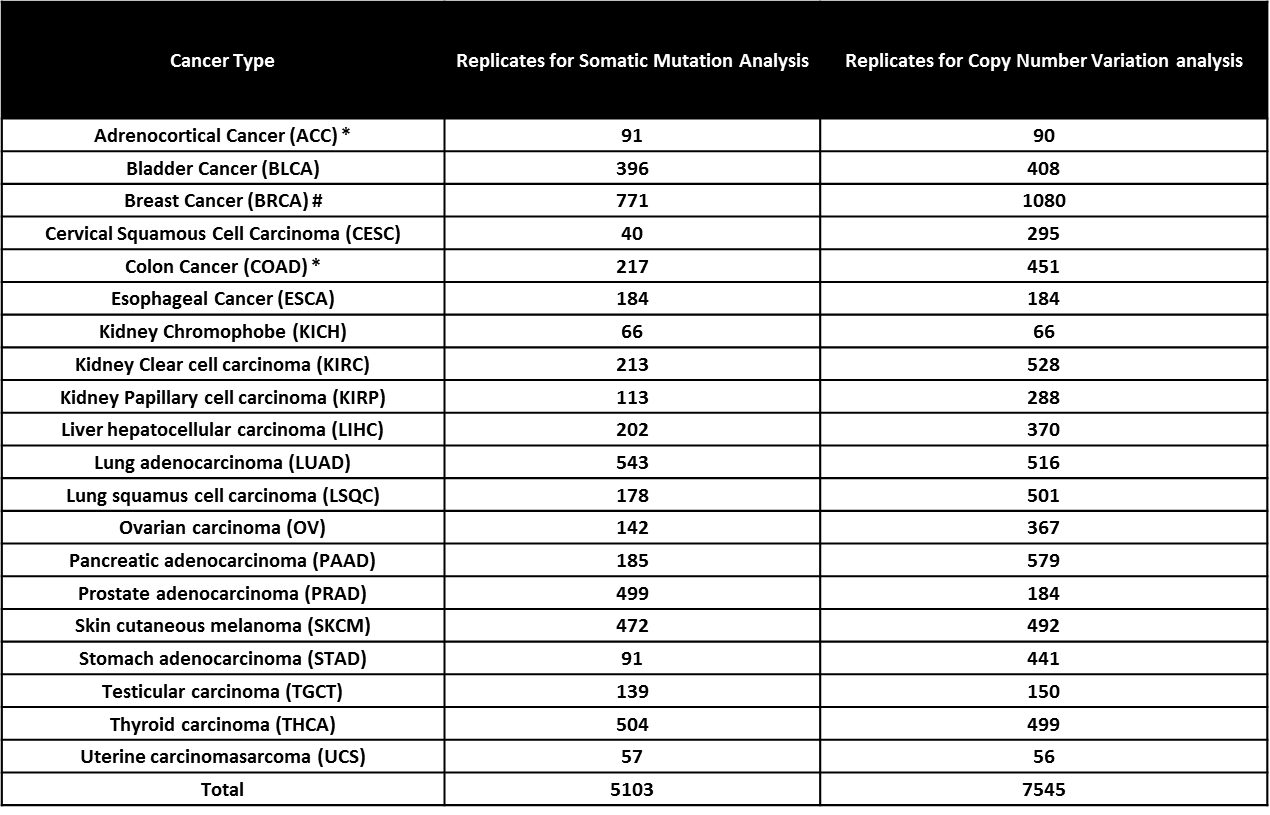
*
