# Supplementary material for: GPCRs show widespread differential mRNA expression and frequent mutation and copy number variation in solid tumors
Source: PLoS Biol. 2019 Nov 25;17(11):e3000434. doi: 10.1371/journal.pbio.3000434 (PMC6901242; doi:10.1371/journal.pbio.3000434)
Supplement: S4 Table — COAD tumors in the sigmoid and transverse colon were compared to normal tissue from those regions. For esophageal tumors (adenocarcinomas and squamous cell carcinomas), DE analysis was compared to esophageal mucosal tissue; data in GTEx for esophageal muscularis tissue were not used in this analysis. Data for sun-exposed skin were compared to melanomas; similar DE results were found if non–sun-exposed skin (in GTEx) was used. Only 9 of 952 TCGA BRCA samples for which gender was recorded were from males, therefore we only used GTEx breast tissue from females as reference normal tissue for DE analysis of BRCA. (DOCX) [file pbio.3000434.s017.docx]

**S4Table**. **The normal tissue types and number of replicates (GTEx database) used for differential expression (DE) analysis of RNA-seq data in normal tissue compared to tumors (TCGA)**. Colonic adenocarcinoma (COAD) tumors in the sigmoid and transverse colon were compared with normal tissue from those regions. For esophageal tumors (adenocarcinomas and squamous cell carcinomas), DE analysis was compared to esophageal mucosal tissue; data in GTEx for esophageal muscularis tissue were not used in this analysis. Data for sun-exposed skin were compared to melanomas; similar DE results were found if non-sun exposed skin (in GTEx) was used. Only 9 of 952 TCGA BRCA samples for which gender was recorded were from males, hence we only used GTEx breast tissue from females as reference normal tissue for DE analysis of BRCA.

*
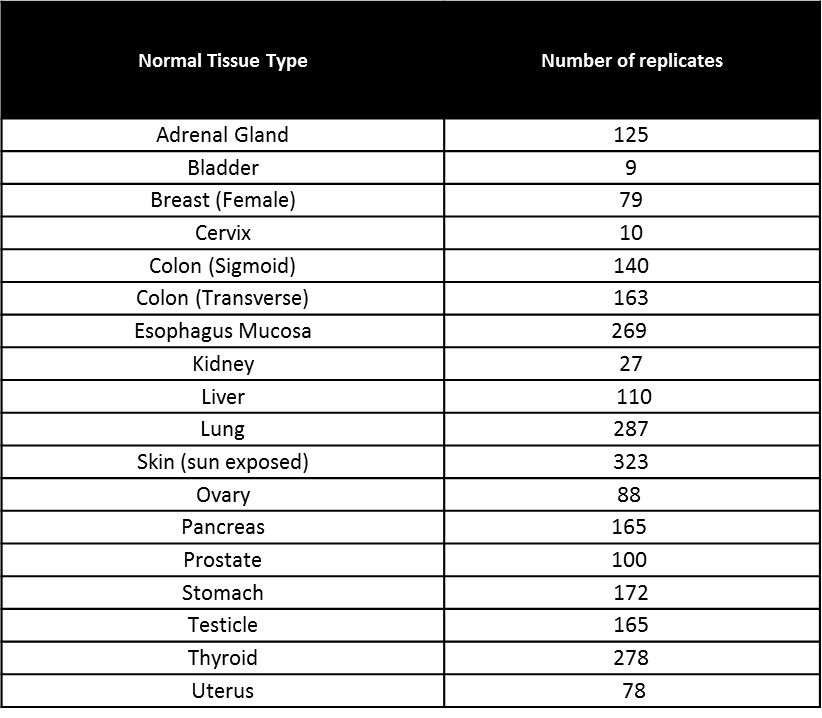
*
