# Supplementary material for: GPCRs show widespread differential mRNA expression and frequent mutation and copy number variation in solid tumors
Source: PLoS Biol. 2019 Nov 25;17(11):e3000434. doi: 10.1371/journal.pbio.3000434 (PMC6901242; doi:10.1371/journal.pbio.3000434)

**S5Table.** **The total number of different types of somatic mutational events for the 30 most frequently mutated GPCRs in the TCGA tumors surveyed**. A complete list is provided as downloadable supplemental material at *insellab.github.io*. In addition, a breakdown of numbers of mutation events for each GPCR in each tumor type is provided in **S1Table**.


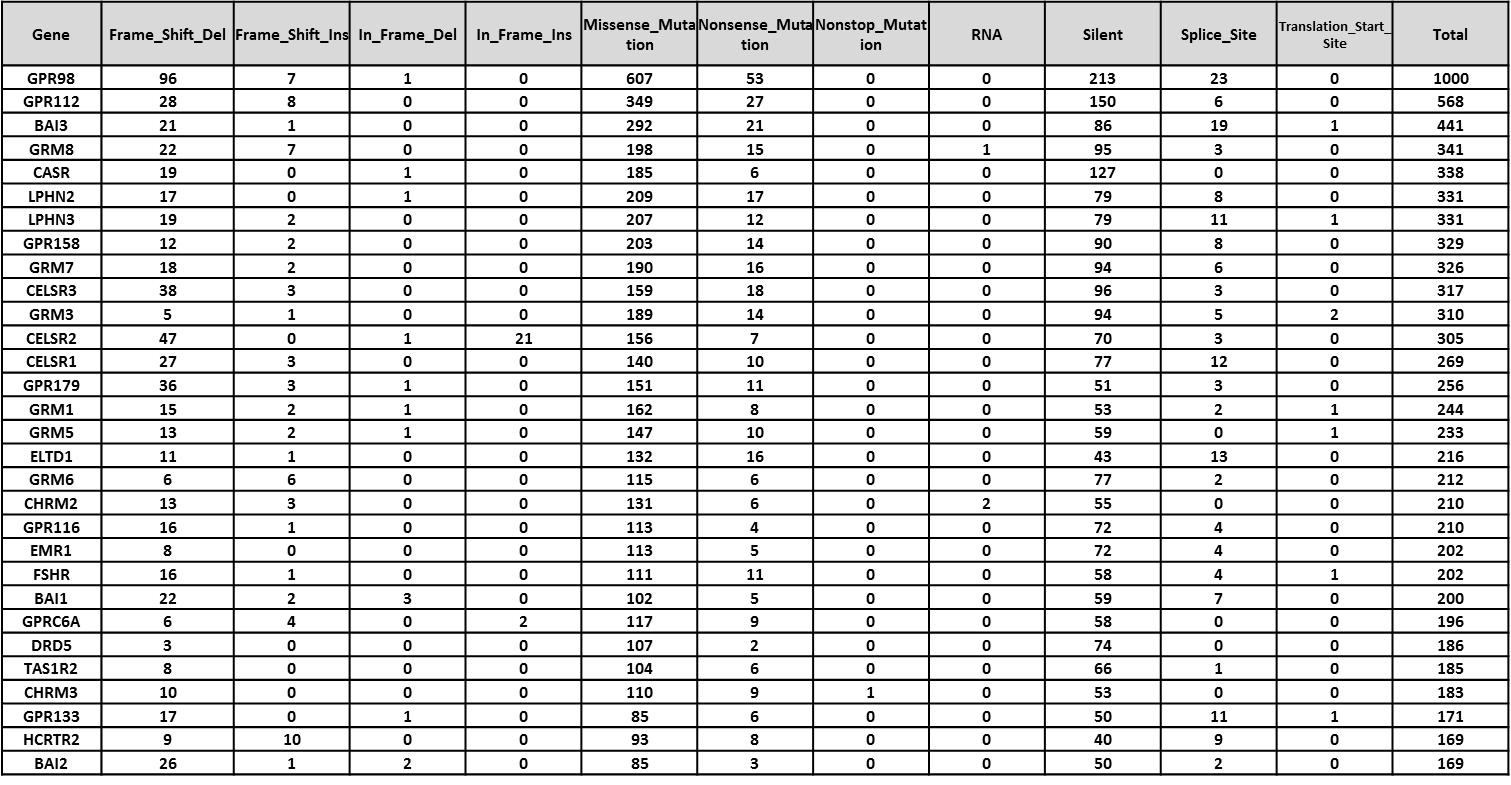

Supplement: S5 Table — A complete list is provided as downloadable supplemental material at insellab.github.io. In addition, a breakdown of numbers of mutation events for each GPCR in each tumor type is provided in S1 Table. (DOCX) [file pbio.3000434.s018.docx]
