# Supplementary material for: GPCRs show widespread differential mRNA expression and frequent mutation and copy number variation in solid tumors
Source: PLoS Biol. 2019 Nov 25;17(11):e3000434. doi: 10.1371/journal.pbio.3000434 (PMC6901242; doi:10.1371/journal.pbio.3000434)

**S6Table. GPCRs showing DE in multiple tumor types.** (**Left**) **Multiple GPCRs have increased expression in multiple types of cancers**. The 25 most widely/commonly overexpressed (OE) GPCRs are listed along with the number of cancer types in which they are OE. (**Center)** **The same tabulation for the 25 most commonly OE GPCRs that are targets for approved drugs**. (**Right**) **The 25 GPCRs most frequently reduced in expression in tumors compared to normal tissue**. GPCRs were considered to have DE if fold-changes were >2, FDR <0.05 and median expression in tumors was >1 TPM. Details of the tumor types where such DE occurs, the size of fold-changes etc. are available in **S2 Table** and at *insellab.github.io*.

A list of GPCRs that are targets for approved drugs was obtained by querying the IUPHAR and CHEMBL databases for lists of approved drugs and the genes that they target. Most such GPCRs are targets for drugs approved by the FDA. The resulting list of ‘druggable’ GPCRs (and the approving agency) is provided in **S2 Table**.

**Overexpressed GPCRs Overexpressed GPCR drug targets Down-regulated GPCRs**


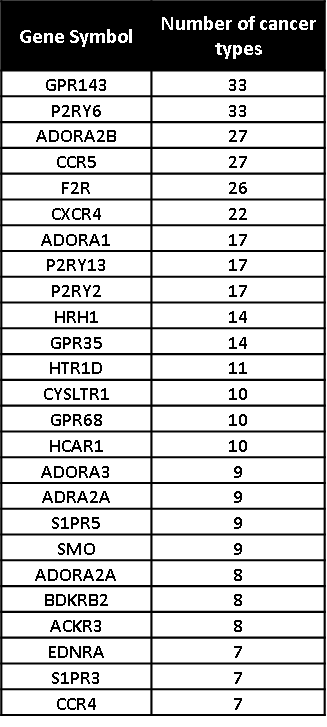

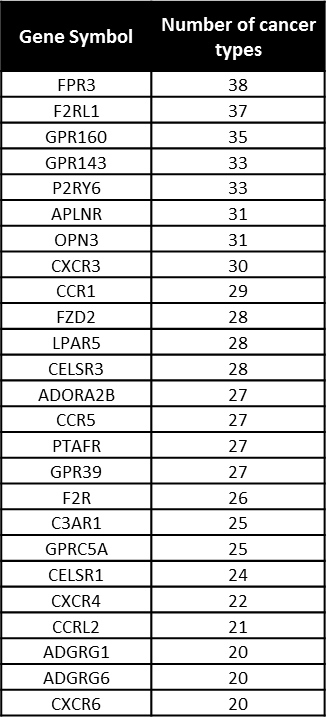

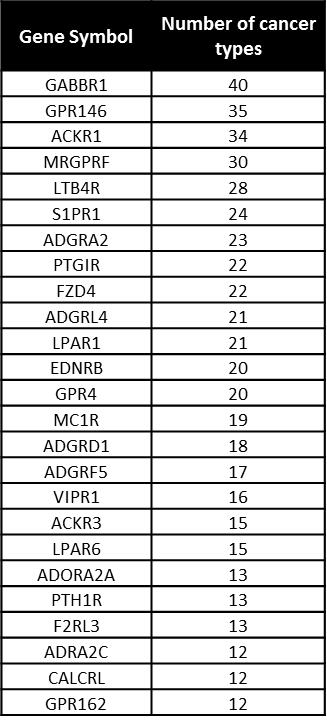

Supplement: S6 Table — (Left) Multiple GPCRs have increased expression in multiple types of cancers. The 25 most widely/commonly OE GPCRs are listed along with the number of cancer types in which they are OE. (Center) The same tabulation for the 25 most commonly OE GPCRs that are targets for approved drugs. (Right) The 25 GPCRs most frequently reduced in expression in tumors compared to normal tissue. GPCRs were considered to have DE if fold-changes were >2, FDR < 0.05, and median expression in tumors was >1 TPM. Details of the tumor types in which such DE occurs, the size of fold-changes, etc., are available in S2 Table and at insellab.github.io. A list of GPCRs that are targets for approved drugs was obtained by querying the IUPHAR and CHEMBL databases for lists of approved drugs and the genes that they target. Most such GPCRs are targets for drugs approved by the FDA. The resulting list of “druggable” GPCRs is provided in S2 Table. OE, overexpressed. (DOCX) [file pbio.3000434.s019.docx]
