# Supplementary material for: GPCRs show widespread differential mRNA expression and frequent mutation and copy number variation in solid tumors
Source: PLoS Biol. 2019 Nov 25;17(11):e3000434. doi: 10.1371/journal.pbio.3000434 (PMC6901242; doi:10.1371/journal.pbio.3000434)
Supplement: S7 Table — (Left) The 30 most frequently overexpressed GPCRs across the 45 different types/subtypes of cancer profiled, along with the number of TCGA tumors (of 5,103 total) in which they have somatic, nonsilent mutations. (Right) The same data, sorted for the 30 GPCRs mutated in the highest number of tumors. Among the most frequently mutated GPCRs, the CELSR genes are also frequently overexpressed, but most other frequently mutated genes are not. (DOCX) [file pbio.3000434.s020.docx]

**S7Table. GPCRs that show frequent overexpression typically show infrequent mutation and vice versa.** (**Left**) The 30 most frequently overexpressed GPCRs across the 45 different types/subtypes of cancer profiled, along with the number of TCGA tumors (of 5103 total) in which they have somatic, non-silent mutations. (**Right**) The same data, sorted for the 30 GPCRs mutated in the highest number of tumors. Among the most frequently mutated GPCRs, the *CELSR* genes are also frequently overexpressed, but most other frequently mutated genes are not.

**
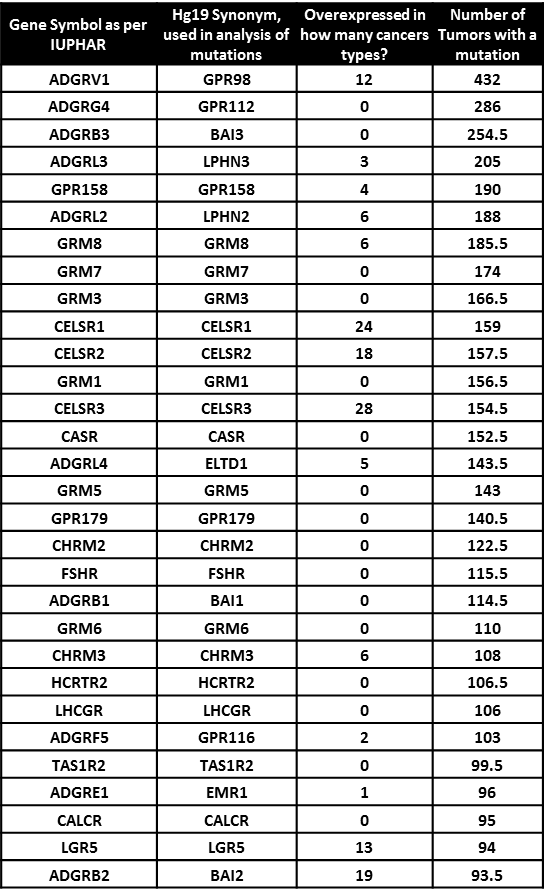

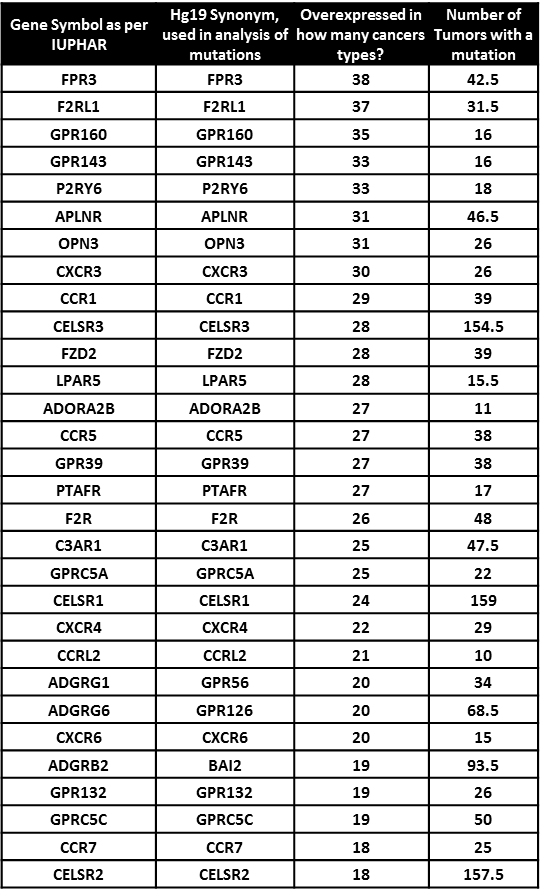
**
