# Supplementary material for: GPCRs show widespread differential mRNA expression and frequent mutation and copy number variation in solid tumors
Source: PLoS Biol. 2019 Nov 25;17(11):e3000434. doi: 10.1371/journal.pbio.3000434 (PMC6901242; doi:10.1371/journal.pbio.3000434)
Supplement: S1 Text — (DOCX) [file pbio.3000434.s021.docx]

**Supporting Text: S1 Text. Concordance of mRNA and protein expression**

Multiple recent studies, exemplified by citations below, support the notion that mRNA expression broadly predicts protein expression and that mRNA expression accounts for the majority of protein expression, compared to other factors such as translational regulation.

Using a yeast model, Csardi et al. [53] have suggested that mRNA expression is highly predictive of steady-state protein abundance. Applying noise-correction techniques to normalize mRNA and protein expression data, this study reported that mRNA accounts for ~85% of steady-state protein abundance. Similarly, [58] showed that in a frog egg model, if dynamics of protein decay and synthesis are accounted for, mRNA expression is highly predictive of protein expression. Data in *E. coli* [55] also suggest that mRNA expression is the primary determinant of protein expression, explaining a larger degree of protein expression than variations in translation.

Further, a study using a drug-treated ovarian cancer mouse xenograft model [56] showed that differentially expressed genes show concordance with changes in protein abundance. Accounting for gene-specific corrections, [54] showed that mRNA abundance is highly predictive of protein abundance in human tissue. Abundances of individual genes and their corresponding proteins correlated with one another by gene-specific proportionality coefficients that were largely independent of tissue and cell type. This result implies a linear, proportional relationship between mRNA and protein, such that changes in mRNA expression will (in general) be expected to predict similar changes in protein expression.

Factors such as those above likely account for much of the discrepancy between gene and protein expression noted in prior studies (e.g., [59]). Re-examination by [57] of the protein abundance data in the latter study (with corrected protein abundance estimates via alternate normalization strategies, using a range of housekeeping proteins) suggested that mRNA accounts for at least 56% of protein abundance and perhaps as high as 80%. The recent studies highlighted above lead us to conclude that data in the current study on differential gene expression likely predict changes in abundance of GPCRs at the protein level in tumors.
