# Supplementary material for: GPCRs show widespread differential mRNA expression and frequent mutation and copy number variation in solid tumors
Source: PLoS Biol. 2019 Nov 25;17(11):e3000434. doi: 10.1371/journal.pbio.3000434 (PMC6901242; doi:10.1371/journal.pbio.3000434)
Supplement: S2 Text — (DOCX) [file pbio.3000434.s022.docx]

**Supporting Text: S2 Text. Correspondence of mRNA and protein expression of *GPRC5A***

The Human Protein Atlas (HPA) ([www.proteinatlas.org](http://www.proteinatlas.org)) provides immunostaining data of normal tissue and tumors, quantifying protein abundance empirically as ‘high’, ‘medium’, ‘low’ and ‘not detected’. A principal challenge in quantifying GPCR protein abundance is the paucity of specific/selective, well-validated antibodies. The difficulties in GPCR protein detection underline the need for positive and negative controls (such as knockdowns/knockouts and overexpressing cells or tissues) for antibody validation. GPCRs are generally low-abundance proteins, making their detection challenging by proteomic methods.

Of GPCRs widely overexpressed in tumors (**S6** **Table**) that were also tested in HPA, we found that only GPRC5A currently had an antibody validated using controls indicated above (Atlas Antibodies, Cat#HPA007928). This antibody was also independently tested and validated in studies on pancreatic cancer cell lines [12], including knockdowns of *GPRC5A*. We thus compared protein abundance in normal and tumor tissue from HPA data that uses this well-validated antibody compared to our estimates for *GPRC5A* mRNA abundance.

Normal tissue shows low to moderate mRNA expression of *GPRC5A* except in lung tissue, in which this GPCR is highly expressed (**S11A** **Fig**), a result in agreement with antibody staining data from HPA (**S11B Fig**). Moreover, staining of multiple tumor replicates (**S10D Fig**) indicates a high frequency of positive staining in pancreatic, colon, stomach, cervical and lung tumors, consistent with *GPRC5A* mRNA expression (**S11C Fig**). Broadly stated, transcriptomic analysis suggested that GPRC5A is generally low-expressed in normal tissue besides the lung, but is widely expressed in a range of tumors. This same general pattern is reflected by *GPRC5A* protein abundance from HPA.
